# Supplementary material for: Cohort profile: the U-BIRTH study on peripartum depression and child development in Sweden
Source: BMJ Open. 2023 Nov 10;13(11):e072839. doi: 10.1136/bmjopen-2023-072839 (PMC10649626; doi:10.1136/bmjopen-2023-072839)
Supplement: Supplementary data [file bmjopen-2023-072839supp001.pdf]

Supplementary Table 1. All assessment tools included in the BASIC and the U-BIRTH studies

Domain of measure	Gestational week 16–18	Gestational week 32	Child-birth	Postpartum 6 weeks	Postpartum 6 months	Postpartum 12 months	Postpartum 18 months	Postpartum 6 years	Postpartum 11 years
	**Data collected from the BASIC project (all completed)**						**Data collected from the U-BIRTH project (Data from 18 months are completed; data collection for 6 and 11 years is ongoing)**		
**Maternal data**									
Depressive symptoms	EPDS<br>DSRS<br>History of depression	EPDS	EPDS	EPDS<br>DSRS (2011–)	EPDS<br>DSRS (2011–)	Single item “how are you”?	DSRS	PHQ-9	PHQ-9<br>DSM-5 Self-Rated Level 1 CCSM
Partner’s depressive symptoms				Single item “partner’s mental health”					
Anxiety symptoms	BAI (2013–)<br>EPDS†<br>STAI-S (2011–2012)	BAI (2013–)<br>EPDS†<br>STAI-S(2011–2012)<br>STAI-T (2011–2012)		BAI (2013–)<br>EPDS†<br>STAI-S (2011–2012)	BAI (2013–)<br>EPDS†<br>STAI-S (2011–2012)<br>STAI-T (2011–2012)				
Trauma and stressful events	Intimate partner violence*			SLE<br>SQ-PTSD (–2011)	SLE<br>SQ-PTSD (2011–)	LITE (2013–)	SLE<br>SPSQ	New SLE	New SLE
Obstetric and gynecological information	Planning of pregnancy<br>Nausea/vomiting<br>Premenstrual symptoms	Pregnancy complications<br>Fear of delivery<br>Delivery expectation		Delivery information<br>Delivery experience					
Medical information	Depression history*<br>Prior contact with psychiatry*<br>Somatic health conditions*<br>Prior and current medication*	Current medication*<br>RLS			Current medication*	Current medication* (2013–)	Current medication*<br>Medical journal	Current medication*<br>Medical journal	Current medication*<br>Medical journal
Lifestyle	BMI before pregnancy*<br>Alcohol*<br>Smoking*<br>Tobacco*	BMI<br>Diet*<br>Alcohol*<br>Smoking*<br>Tobacco*		BMI<br>Smoking*<br>Alcohol*<br>Tobacco*	Smoking*<br>Alcohol*<br>Tobacco*		Smoking*<br>Alcohol*<br>Tobacco*	Smoking*<br>Alcohol*<br>Tobacco*	Smoking*<br>Alcohol*<br>Tobacco*

Supplementary Table 1. (Continued)

Domain of measure	Gestational week 16–18	Gestational week 32	Child-birth	Postpartum 6 weeks	Postpartum 6 months	Postpartum 12 months	Postpartum 18 months	Postpartum 6 years	Postpartum 11 years
Sleep	Sleeping habits before pregnancy*<br>Hours of sleep during pregnancy*<br>Sleep quality (2018–)*	Sleeping problems*<br>Hours of sleep during pregnancy*<br>Sleep quality (2018–)*		Sleep quality*	Sleep quality*	Hours of sleep*	Sleep quality*	Sleep quality*	Sleep quality*
Personality and resilience measures		ASQ<br>SSP (–2011)<br>SOC-29 (2013–)<br>RS-14 (2013–)			PBQ	VPSQ (2013–)			SOC
Others	Sociodemographic information: age, , country of birth, education, employment*	Sociodemographic information: employment*		Breastfeeding*<br>General questions*<br>Life right now*<br>Partner support*<br>Social support from others*	Breastfeeding*<br>General questions*<br>Life right now*Marital status* Partner support*	Breastfeeding*<br>General questions*<br>Life right now (2013–)*	CHAOS<br>Chores and activity*<br>Life right now*<br>PSS<br>SF-36<br>Second parent's status*<br>Social life*	Another pregnancy*<br>CHAOS<br>Chores and activities*Life right now*<br>Maternal leave*<br>Partner's status*<br>Previous breastfeeding*<br>PSI<br>SF-36<br>Social life*<br>Sociodemographic information*	CHAOS<br>EQ-5<br>PSQ<br>Sociodemographic information*
**Child outcome assessments**									
General measure			Apgar score 1 min<br>Apgar score 5 min<br>Birthweight<br>Head circumference<br>Body length<br>NICU and birth complications				Child's growth, traits, and behavior*; general Screening†	Child's growth, traits, and behavior*; general Screening†	Child's growth, traits, and behavior*; general Screening†
Sleep				Sleeping habits and problems*	Sleeping habits and problems*	Sleeping habits and problems*	Sleeping habits and problems*		

Supplementary Table 1. (Continued)

Domain of measure	Gestational week 16–18	Gestational week 32	Child-birth	Postpartum 6 weeks	Postpartum 6 months	Postpartum 12 months	Postpartum 18 months	Postpartum 6 years	Postpartum 11 years
**Child outcome assessments**									
Social-emotional development				Interest in face<br>Eye contact<br>Social smile<br>General mood			Relationship with parents*	Child's social skills*	Child's social skills and relationship*
Behavior and temperament				IBQ (2013 –)		IBQ (2013 –)	CBCL (1.5); ECBQ	CBCL (1.5-5)	CBCL (6-18)
Language development							CBCL-LDS<br>General Screening†	Child's language development*<br>General Screening†	Child's language health*
Neuropsychological development								FTF	FTF
School							Participation in preschool (parent-report)*	School performance (parent-report)*	Child's school performance (parent-report* and register data††)
Biological sample							Saliva sample	Saliva sample	
Others							Free time activities*	Child's chores*; child's free-time*	Child's chores*; child's free-time*

ASQ: Attachment Style Questionnaire; BAI: Beck Anxiety Inventory; CBCL: Child Behavior Checklist; CBCL-LDS: Child Behavior Checklist, Language Development Survey; CCSM: Cross-Cutting Symptom measure, DSM-5; CHAOS: Confusion, Hubbub and Order Scale ; DSRS: Depression Self-Rating Scale; DSM-5: the Diagnostic and Statistical Manual of Mental Disorders, the 5th Edition; ECBQ: Early Childhood Behavior Questionnaire; EPDS: Edinburgh Postnatal Depression Scale; EQ-5D: Health-Related Quality of Life Questionnaire; FTF: Five to Fifteen; IBQ: Infant Behavioral Questionnaire; LITE: Lifetime Incidence of Traumatic Events; PBQ: Postpartum Bonding Questionnaire; PHQ9: Patient Health Questionnaire; PSI: Parenting Stress Index; PSQ: Parenting Style Questionnaire; PSS: Perceived Stress Scale; RLS: Restless legs questionnaire; RS-14: Resilience Scale; SF-36: The 36-Item Short Form Health Survey; SLE: Stressful Life Events Scale; SOC-29: Sense of Coherence; SPSQ: Swedish Parenthood Stress Questionnaire; SQ-PTSD: Screen Questionnaire-Post-Traumatic Stress Disorder; SSP: Swedish Universities Scales of Personality; STAI-S: State-Trait Anxiety Inventory-State Scale; STAI-T: State-Trait Anxiety Inventory-Trait Scale; VPSQ: Vulnerable Personality Style Questionnaire. Period of usage in the BASIC is stated in parentheses if other than 2009–2017. *Questions designed by the research team. †The Edinburgh Postnatal Depression subscales for detecting anxiety.
