# Supplementary material for: Cohort profile: the U-BIRTH study on peripartum depression and child development in Sweden
Source: BMJ Open. 2023 Nov 10;13(11):e072839. doi: 10.1136/bmjopen-2023-072839 (PMC10649626; doi:10.1136/bmjopen-2023-072839)
Supplement: Author's manuscript [file bmjopen-2023-072839.draft_revisions.pdf]

BMJ Open is committed to open peer review. As part of this commitment we make the peer review history of every article we publish publicly available.

When an article is published we post the peer reviewers' comments and the authors' responses online. We also post the versions of the paper that were used during peer review. These are the versions that the peer review comments apply to.

The versions of the paper that follow are the versions that were submitted during the peer review process. They are not the versions of record or the final published versions. They should not be cited or distributed as the published version of this manuscript.

BMJ Open is an open access journal and the full, final, typeset and author-corrected version of record of the manuscript is available on our site with no access controls, subscription charges or pay-per-view fees (http://bmjopen.bmj.com).

If you have any questions on BMJ Open's open peer review process please email info.bmjopen@bmj.com

BMJ Open

Cohort profile: the U-BIRTH study on perinatal depression and child development in Sweden

Journal:	*BMJ Open*
Manuscript ID	bmjopen-2023-072839
Article Type:	Cohort profile
Date Submitted by the Author:	16-Feb-2023
Complete List of Authors:	Tu, Hsing-Fen; Uppsala University, Department of Women's and Children's Health; Uppsala University, Department of Psychology<br>Fransson, Emma; Uppsala University, Department of Women's and Children's Health; Karolinska Institutet, Centre for Translational Microbiome Research, Department of Microbiology, Tumor and Cell Biology<br>Kunovac Kallak, Theodora; Uppsala University, Department of Women's and Children's Health<br>Elofsson, Ulf; Uppsala University, Department of Women's and Children's Health<br>Ramklint, Mia; Uppsala University, Department of Neuroscience, Psychiatry<br>Skalkidou, Alkistis; Uppsala University, Department of Women's and Children's Health
Keywords:	GYNAECOLOGY, PSYCHIATRY, Community child health < PAEDIATRICS

SCHOLARONE™
Manuscripts

I, the Submitting Author has the right to grant and does grant on behalf of all authors of the Work (as defined in the below author licence), an exclusive licence and/or a non-exclusive licence for contributions from authors who are: i) UK Crown employees; ii) where BMJ has agreed a CC-BY licence shall apply, and/or iii) in accordance with the terms applicable for US Federal Government officers or employees acting as part of their official duties; on a worldwide, perpetual, irrevocable, royalty-free basis to BMJ Publishing Group Ltd ("BMJ") its licensees and where the relevant Journal is co-owned by BMJ to the co-owners of the Journal, to publish the Work in this journal and any other BMJ products and to exploit all rights, as set out in our licence.

The Submitting Author accepts and understands that any supply made under these terms is made by BMJ to the Submitting Author unless you are acting as an employee on behalf of your employer or a postgraduate student of an affiliated institution which is paying any applicable article publishing charge ("APC") for Open Access articles. Where the Submitting Author wishes to make the Work available on an Open Access basis (and intends to pay the relevant APC), the terms of reuse of such Open Access shall be governed by a Creative Commons licence – details of these licences and which Creative Commons licence will apply to this Work are set out in our licence referred to above.

Other than as permitted in any relevant BMJ Author's Self Archiving Policies, I confirm this Work has not been accepted for publication elsewhere, is not being considered for publication elsewhere and does not duplicate material already published. I confirm all authors consent to publication of this Work and authorise the granting of this licence.

For peer review only

Cohort profile: the U-BIRTH study on perinatal depression and child development in Sweden

Hsing-Fen Tu[1,2], Emma Fransson[1,3], Theodora Kunovac Kallak[1], Ulf Elofsson[1], Mia Ramklint[4], Alkistis Skalkidou[1*]
Affiliations
[1]Department of Women's and Children's Health, Uppsala University, Uppsala, Sweden
[2]Department of Psychology, Uppsala University, Uppsala, Sweden
[3]Centre for Translational Microbiome Research, Department of Microbiology, Tumor and Cell Biology, Karolinska Institutet, Stockholm, Sweden
[4]Department of Neuroscience, Psychiatry, Uppsala University, Uppsala, Sweden
[*]Correspondence: alkistis.skalkidou@kbh.uu.se

For peer review only

ABSTRACT

Purpose
The current U-BIRTH cohort extends our previous cohort Biology, Affect, Stress, Imaging and Cognition (BASIC), assessing the development of children up to 11 years after birth. The U-BIRTH study aims to (1) assess the impact of exposure to perinatal mental illness on the children´s development taking into account biological and environmental factors during intrauterine life and childhood; (2) identify early predictors of child neurodevelopmental and psychological problems using biophysiological, psychosocial, and environmental variables available during pregnancy and early postpartum.

Participants
All mothers participating in the previous BASIC cohort are invited, and mother-child dyads recruited in the U-BIRTH project are consecutively invited to questionnaire assessments and biological sampling when the child is 18 months, 6 years, and 11 years old. Data collection for 18 months has been completed for 2882 families, while the other two assessments are ongoing.

Findings to date
Based on the complete data from pregnancy to 18 months postpartum, perinatal mental health was significantly associated with the development of attentional control and gaze-following behaviours, which are critical to cognitive and social learning later in life. Moreover, infants of depressed mothers had an elevated risk of difficult temperament and behavioural problems compared to infants of non-depressed mothers. Analyses of biological samples showed that perinatal depression and anxiety were related to DNA methylation differences in infants. However, there were no methylation differences in relation to infants' behavioural problems at 18 months of age.

Future plans
Given that the data collection at 18 months is completed, analyses are now being undertaken. Currently, assessments for children that have reached 6 and 11 years are ongoing.

Strengths and limitations

- This population-based study provides rich data across pregnancy, infancy, early childhood, and the pre-teen periods. The sample corresponds to one of the largest cohorts within the Scandinavian countries.
- To disentangle how the complex interactions between maternal well-being and health, perinatal exposures, and childhood environment contribute to the cognitive, social and

For peer review only - http://bmjopen.bmj.com/site/about/guidelines.xhtml

emotional development of the child, this study includes data from biological, psychosocial, and behavioural assessments using a longitudinal design.

- The cohort's representability is relatively low at about 10% of the background population, impacting on the generalizability of the findings.
- Though the assessments of child development included in the project are well accepted and widely applied in the field, most of them rely mainly on parent-report questionnaires. This could introduce information bias.

INTRODUCTION

Perinatal depression is a significant complication that affects many women during pregnancy and/or the first year postpartum (1). Almost one in every seven pregnancies experience perinatal depression (2-4) Moreover, perinatal depression increases the risk of maternal self-harm and suicidal acts (5, 6). While perinatal depression affects many mothers and their families' daily life (7), it also profoundly affects offspring's lifelong development (8). Growing evidence suggests that the intrauterine foetal and neonatal distress increase the risk of substantial long-term harm on physical, neurological, and cognitive development (9). The underlying mechanisms might result from the interplay between perinatal environment and the process of epigenetic modification (10).

An extensive body of work has shown that perinatal depression not only affects the mother's daily life but also elevates the risk of the children for poor self-regulation (11), delayed executive functions (12), and neurodevelopmental disorders, such as attention deficit hyperactivity disorder and autism spectrum disorder (13, 14). While mothers with perinatal depression might continue to develop chronic depression (15), increasing evidence shows that children of depressed mothers are at higher risk to develop adjustment problems, behavioural problems, or mood disorders and might have more difficulties in social, cognitive, and academic functioning compared to children of non-depressed mothers (16-19). Though associations between perinatal mental health and children's outcome are well-documented, the underlying mechanisms remain unclear. One important aspect is that the high degree of complexity of both maternal mental health and child development makes it challenging to study. Especially, the complexity is deeply embedded in the temporal, social, cultural, biological, genetic, and environmental factors, etc. Recent research shows that the trajectories of maternal depression and children's developmental trajectories are both non-linear (15, 20). Besides, high comorbidity between depression, anxiety, and stress is common (21), and how different symptoms develop over time and affect children's outcomes is still inconclusive. Yet, research focusing on the heterogeneity of perinatal depression and how it affects offspring's development is still rare (22).

Taken together, not only multivariate and longitudinal research is needed in the field, but it is also essential to cover a wide aspect of assessments, including biophysiological, psychosocial, and environmental factors of mothers. This approach will hopefully provide a deeper understanding of the associations between maternal mental health and the developmental trajectories of children.

In the current U-BIRTH protocol, we extend our previous large cohort, Biology, Affect, Stress, Imaging and Cognition (BASIC) (23), including 5492 mothers from pregnancy to a year postpartum; the U-BIRTH project focuses on the follow-up of the infants, and continues to explore the full path of human development from foetus to pre-teen. Based on notions from life course epidemiology, and the importance of early life factors regarding patterns impacting long term health, this project sets out to study exposure to different trajectories of maternal

For peer review only - http://bmjopen.bmj.com/site/about/guidelines.xhtml

depression; in utero and or after birth on children´s social and emotional development. Furthermore, factors of resilience and increased risk of atypical development are investigated. We aim at disentangling the complex pathways between maternal depression and children's outcomes by mapping a wide range of psychological and biophysiological factors from the longitudinal dataset of mothers up to their children are at the age of 6 and 11 years. More specifically, we intend to explore the complex cross-generational transmission pathways based on a multi-level approach, from the molecular level to the psychosocial level. The U-BIRTH study thus aims to (1) assess the impact of exposure to perinatal mental illness on the children´s development taking into account biological and environmental factors during intrauterine life and childhood; (2) identify early predictors of child neurodevelopmental and psychological problems using biophysiological, psychosocial, and environmental variables available during pregnancy and early postpartum. Specific objectives of the current protocol include: (1) presenting a detailed description of the U-BIRTH project, a continuation of the BASIC cohort with follow-ups up to 11 years after delivery, and (2) giving updated summaries of the previous findings of associations between perinatal mental health and offspring's outcomes from both BASIC and U-BIRTH projects. Ultimately, we hope to identify predictors as well as risk- and protective factors that might support future prevention and intervention programs.

COHORT DESCRIPTION

Study design

U-BIRTH, the follow-up project of the BASIC study (23) is hosted at the Department of Obstetrics and Gynaecology at Uppsala University Hospital. In the BASIC project, 5492 women (17.3% of all), participated between September 2009 to November 2018. Participants were followed-up at multiple points from gestational week 16–18 through 12 months postpartum. In the BASIC project (23), data from a variety of psychometric self-report instruments and biological samples were collected (for more information, please see also https://www.basicstudie.se/). All mother-child dyads who participated in the BASIC project are invited to join the U-BIRTH project (https://www.ubirth.se/) at each assessment. Most of the participants already have data from multiple time points acquired within the BASIC project. The sociodemographic information of participating families (who have sent signed consent), as well as of families in the general population of Uppsala County and Sweden is listed in Table 1. All mother-child dyads recruited in the U-BIRTH project are invited to a series of assessments when the child is at age of 18 months, 6 years, and 11 years. Thus, children who complete the assessments at the age of 11 years will have data from a maximum of seven time points (Table 2). Mothers who complete the assessments when their child reaches 11 years old will have provided data from maximum nine time points (Table 3). Figure 1 illustrates the data timeline included in the protocol. Currently, 3525 families have completed at least one assessment. All children have reached 18 months of age and the data acquisition of this phase is completed. There were 6083 invitations to the study for the 18 months postpartum, 3341 mother-infant dyads (55%) consented to participate. Among those consenting to participate in survey, 88% completed the survey at 18 months postpartum. Seventy-three percent of the children have today reached 6 years of age. There were 4361 invitations sent, and 1720 (39%) mother-child dyads have fully completed the assessments. Moreover, 1547 (25%) children have reached 11 years of age and 683 have completed the assessments. Families not responding to the invitation receive one email reminder. Families who agree to participate but do not answer the survey receive three email reminders. The flowchart for participants is presented in Figure 2.

For peer review only - http://bmjopen.bmj.com/site/about/guidelines.xhtml

Table 1. Sociodemographic information of the U-BIRTH cohort and the general population of Uppsala County and Sweden

	**U-BIRTH (n = 3525*)**			**Uppsala County† (2009–2016, n = 32 258)**	**Sweden† (2009–2016, n = 902698)**
**Variable**	**Available data (n)**	**% or mean**	**Missing, n**	**% or mean**	**% or mean**
Maternal age (mean, sd)	3374	31.8 (±4.4)	151	30.4 (±5.2)	30.3 (±5.3)
Country of origin					
Scandinavia	3069	87.1	227	80.3	76.6
Other countries	229	6.5		19.7	23.3
Education					
≤ 12 years	520	17	471	46.7	48.5
Cohabiting with the 2nd legal guardian	2790	97.2	656	95.7	93.1
BMI before pregnancy kg/m^2	3094	23.7 (±4.2)	431		
< 18.5	93	2.6		2.2	2.9
18.5 ≤ BMI < 25	2146	60.9		58.6	65.6
25 ≤ BMI < 30	594	16.9		25.9	25.3
≥ 30	261	7.4		13.4	13.0
Infant birth weight, gram	3257	3593 (±546.6)	268	3498	3504
Infant birth weight < 2500g	92	2.8 %		5.1	4.4
Infant birth weight ≥ 4500g	142	4.4		3.9	3.5
Primiparous	1547	47.5	265	43.8	43.9
Premature delivery (< week 37)	168	5.2	265	7.4	6.0
Apgar 5 < 7	40	1.2	282	2.0	1.3

BMI: Body mass index. Percentages are given in relation to available data from women.
*Data downloaded on 2023-01-27. At this time points, there were 3525 families already completed at least one time assessment. These children were all born between 2010 and 2019. †Data retrieved on 2023-01-27 from the Swedish Medical Birth Register and Swedish National board of Health and Welfare (www.socialstyrelsen.se).

For peer review only

Table 2. Content of children's outcome measures included in the U-BIRTH project

Domain of measure	Delivery info	6 weeks	6 months	12 months	18 months	6 years	11 years
	Data collected from the BASIC project (all completed)				Data collected from the U-BIRTH project (Data from 18 months are completed; data collection for 6 and 11 years is ongoing)		
General measure	Apgar score 1 min<br>Apgar score 5 min<br>Birthweight<br>Head circumference<br>Body length<br>NICU and birth complications				Child's grow, traits, and behavior*	Child's grow, traits, and behavior*	Child's grow, traits, and behavior*
Sleep		Sleeping habits and problems*	Sleeping habits and problems*	Sleeping habits and problems*	Sleeping habits and problems*		
Social-emotional development		Interest in face<br>Eye contact<br>Social smile<br>General mood			Relationship with parents*	Child's social skills*	Child's social skills and relationship*
Behaviour and temperament		IBQ (2013–)		IBQ (2013–)	CBCL (1.5)<br>ECBQ	CBCL (1.5-5)	CBCL (6-18)
Language development					CBCL-LDS	Child's language development*<br>General Screening†	Child's language health*
Neuro-development					FTF	FTF	FTF
School					Participation in preschool (parent-report)*	School performance (parent-report)*	Child's school performance (parent-report* and data from the Swedish National Agency for Education††)
Biological sample					Saliva sample	Saliva sample	
Others			Food or diet*		Free time activities*	Child's chores*<br>Child's free-time*	Child's chores*<br>Child's free-time*

CBCL: Child Behavior Checklist; CBCL-LDS: Child Behavior Checklist, Language Development Survey; ECBQ: Early Childhood Behavior Questionnaire; FTF: Five to Fifteen; IBQ: Infant Behavioral Questionnaire; SLE: Stressful Life Events Scale. Period of usage in the BASIC is stated in parentheses if other than 2009–2017. *Questions designed by the research team. †Data source: the National Board of Health and Welfare https://www.socialstyrelsen.se/. Information includes the records related to referral and visit to a speech therapist at age of 2, 3, 4, and 5 years. †† Data source: the Swedish National Agency for Education (skolverket) https://www.skolverket.se/skolutveckling/statistik.

For peer review only - http://bmjopen.bmj.com/site/about/guidelines.xhtml

Table 3. Content of maternal surveys included in U-BIRTH project

Domain of measure	Gestational week 16–18	Gestational week 32	Postpartum 6 weeks	Postpartum 6 months	Postpartum 12 months	Postpartum 18 months	Postpartum 6 years	Postpartum 11 years
	Data collected from the BASIC project (all completed)					Data collected from the U-BIRTH project (Data from 18 months are completed; data collection for 6 and 11 years is ongoing)		
Depressive symptoms	EPDS<br>DSRS<br>History of depression	EPDS	EPDS<br>DSRS (2011–)	EPDS<br>DSRS (2011–)	Single item "how are you"?	DSRS	PHQ-9 (MINI)	PHQ-9 (MINI)
Partner's depressive symptoms			Single item "partner's mental health"					
Anxiety symptoms	BAI (2013–)<br>EPDS†<br>STAI-S (2011–2012)	BAI (2013–)<br>EPDS†<br>STAI-S(2011–2012)<br>STAI-T (2011–2012)	BAI (2013–)<br>EPDS†<br>STAI-S (2011–2012)	BAI (2013–)<br>EPDS†<br>STAI-S (2011–2012)<br>STAI-T (2011–2012)				
Trauma and stressful events	Intimate partner violence*		SLE<br>SQ-PTSD (–2011)	SLE<br>SQ-PTSD (2011–)	LITE (2013–)	SLE<br>SPSQ	New SLE	New SLE
Obstetric and gynecological information	Planning of pregnancy<br>Nausea/vomiting<br>Premenstrual symptoms	Pregnancy complications<br>Fear of delivery<br>Delivery expectation	Delivery information<br>Delivery experience					
Medical information	Depression history*<br>Prior contact with psychiatry*<br>Somatic health conditions*<br>Prior and current medication*	Current medication*<br>RLS		Current medication*	Current medication* (2013–)	Current medication*	Current medication*	Current medication*
Lifestyle	BMI before pregnancy*<br>Alcohol*<br>Smoking*<br>Tobacco*	BMI<br>Diet*<br>Alcohol*<br>Smoking*<br>Tobacco*	BMI<br>Smoking*<br>Alcohol*<br>Tobacco*	Smoking*<br>Alcohol*<br>Tobacco*		Smoking*<br>Alcohol*<br>Tobacco*	Smoking*<br>Alcohol*<br>Tobacco*	Smoking*<br>Alcohol*<br>Tobacco*
Sleep	Sleeping habits before pregnancy*	Sleeping problems*	Sleep quality*	Sleep quality*	Hours of sleep*	Sleep quality*	Sleep quality*	Sleep quality*

For peer review only - http://bmjopen.bmj.com/site/about/guidelines.xhtml

	Hours of sleep during pregnancy*<br>Sleep quality (2018–)*	Hours of sleep during pregnancy*<br>Sleep quality (2018–)*						
Personality and resilience measures		ASQ<br>SSP (–2011)<br>SOC-29 (2013–)<br>RS-14 (2013–)		PBQ	VPSQ (2013–)			SOC
Others	Sociodemographic information: age, , country of birth, education, employment*	Sociodemographic information: employment*	Partner support*<br>Social support from others*<br>Breastfeeding*<br>General questions*<br>Life right now*	Marital status*<br>Partner support*<br>Breastfeeding*<br>General questions*<br>Life right now*	Breastfeeding*<br>General questions*<br>Life right now (2013–)*	CHAOS<br>SF-36<br>Second parent's status*<br>PSI<br>Social life*<br>Chores and activity*<br>Life right now*	Sociodemographic information*<br>CHAOS<br>SF-36<br>Previous breastfeeding*<br>Maternal leave*<br>Another pregnancy*<br>Life right now*<br>Partner's status*<br>PSI<br>Social life*<br>Chores and activities*	Sociodemographic information*<br>CHAOS<br>EQ-5D<br>PSQ<br>SF-36

Note: In one subset of children, maternal EPDS was accessed as one time point when the child was born. This is not listed in the current table.
ASQ: Attachment Style Questionnaire; BAI: Beck Anxiety Inventory; CHAOS: Confusion, Hubbub and Order Scale ; DSRS: Depression Self-Rating Scale; EPDS: Edinburgh Postnatal Depression Scale; EQ-5D: Health-Related Quality of Life Questionnaire; LITE: Lifetime Incidence of Traumatic Events; PBQ: Postpartum Bonding Questionnaire; PHQ9: Patient Health Questionnaire; PSI: Parenting Stress Index; PSQ: Parenting Style Questionnaire; PSI: Parenting Stress Index short form; RLS: Restless legs questionnaire; RS-14: Resilience Scale; SF-36: The 36-Item Short Form Health Survey; SLE: Stressful Life Events Scale; SOC-29: Sense of Coherence; SPSQ: Swedish Parenthood Stress Questionnaire; SQ-PTSD: Screen Questionnaire-Post-Traumatic Stress Disorder; SSP: Swedish Universities Scales of Personality; STAI-S: State-Trait Anxiety Inventory-State Scale; STAI-T: State-Trait Anxiety Inventory-Trait Scale; VPSQ: Vulnerable Personality Style Questionnaire. Period of usage in the BASIC is stated in parentheses if other than 2009–2017.
*Questions designed by the research team. †The Edinburgh Postnatal Depression subscales for detecting anxiety.

For peer review only

For peer review only - http://bmjopen.bmj.com/site/about/guidelines.xhtml

Data collection

The U-BIRTH and BASIC projects contain data from a wide range of perinatal psychometric self-report instruments, various modalities of biological sampling at birth, and data from medical records. The three follow-up timepoints include mother-reported instruments, as well as saliva sampling from the children at 18 months and 6 years of age. For exact measures and timeline please see Table 2 and Figure 1. Maternal psychometric data consist of repeated measures of depressive symptoms (the Edinburgh Postnatal Depression Scale, EPDS (24); and/or the Depression Self-Rating Scale, DSRS (25), anxiety symptoms (the Beck Anxiety Inventory, BAI (26, 27); or the State-Trait Anxiety Inventory for Adult, STAI-AD (28), stressful events (Stressful Life Events Scale, SLE (29)), and post-traumatic stress symptoms (the Screen Questionnaire-Post-Traumatic Stress Disorder, SQ-PTSD (30)). Other maternal psychometric measures include the mother's attachment pattern (the Attachment Style Questionnaire, ASQ (31)), the incidence of traumatic experiences (the Lifetime Incidence of Traumatic Events, LITE (32, 33)), postpartum bonding (the Postpartum Bonding Questionnaire, PBQ (34, 35)), sense of coherence (the Sense of Coherence, SOC-29 (36)), personality (the Swedish Universities Scales of Personality, SSP (37); and the Vulnerable Personality Style Questionnaire, VPSQ (38)), and resilience (the Resilience Scale, RS-14 (39)) are collected only at one-time point. For mothers who consent to provide their medical information, data are retrieved at different time points. Moreover, they are invited to fill out the Patient Health Questionnaire, PHQ-9 MINI (40), twice when their children reach the age of 6 and 11 years. Other repeated measures of the maternal data include lifestyles (e.g. Body Mass Index, diet, consumption of alcohol and tobacco), sleep habits, social environment, quality of life, SF-36 (41), health-related quality of life, EQ-5D (42), home environment (the Confusion, Hubbub, and Order Scale, CHAOS (43)), parenting experiences (the Parenting Stress Index short form, PSI (44); the Swedish Parenthood Stress Questionnaire, SPSQ, (45), and general questions about the living situation, etc. For biological data, as illustrated in Figure 1, maternal blood samples, microbiota samples, and amniotic fluid, umbilical cord biopsy, and foetal blood samples are acquired within the BASIC project at birth. For the description of biological data collection, handling, and storage, please see the BASIC cohort profile (23).

Children's outcome measures include domains such as general physical measures, sleep behaviour, social-emotional development, behaviour, language development, neurodevelopment, school participation and performance, and others (e.g. diet and free time activities, etc.; Table 3). General physical measures include growth data from birth, 18 months, 6 years, and 11 years old. Sleep behaviours measured at the age of 6 weeks, 6 months, 12 months, and 18 months focus on a child's sleep habits or problems. The assessments of social-emotional development include eye contact, social smile, etc. during the first 6 weeks after birth, relationship with parents at the age of 18 months, 6 and 11 years, and stress (the Stressful Life Events Scale) at 11 years old. Children's social skills are measured at 6 and 11 years old. Children's behavioural measures are reported by parents when children are at the age of 6 weeks, 12 months (the Infant Behaviour Questionnaire, IBQ (46)), 18 months (the Early Childhood Behaviour Questionnaire, ECBQ (47), the Child Behaviour Checklist for 1.5–five years old (48)); and 6 years (the Child Behaviour Checklist for 1.5–5 years old (48)) and 11 years (the Child Behaviour Checklist for 6–18 years old (49)). The development of language is measured at 18 months, 6 years (the Language Development Survey (50)) , and 11 years old. For assessment of development, the Five to Fifteen questionnaire, FTF (51, 52), is applied to detect symptoms or problems of attention deficit hyperactivity disorder at the age of 6 and 11 years. Other aspects of daily life such as participation in school, household, and free time activities, etc. are also included in the survey from 18 months, 6, and 11 years old. In addition, for parents who provide consent to link their results to the Swedish National Registers,

For peer review only - http://bmjopen.bmj.com/site/about/guidelines.xhtml

information related to maternal diagnoses, children's diagnoses and information on the growth of the child recorded in the Child Health Centres, referral to medical services, breast feeding information in the first year postpartum, etc. will be also collected for further analysis. In addition, a subset of the participants (n = 120) was assessed from pregnancy to 30 months postpartum in collaboration with the Uppsala Child and Baby Lab at the Department of Psychology at Uppsala University (the BASIC Child Project, for the project descriptions, https://nyu.databrary.org/volume/828, 53). The final samples of this subset at the four assessment points at the age of 6, 10, 18, and 30 months. Data acquisition took place between 2014 and 2018. Infants' cognitive and social-emotional development was assessed using behavioural observations, eye-tracking measures, and parent-reports. Inhibitory control, executive functions, working memories, and parental scaffolding behaviours were evaluated by behavioural observations. Data of this subset will also be included in the final analysis. Hence, some children will provide results from 11 assessment points in total when they reach 11 years old.

Ethical considerations

This research project fulfils General Data Protection Regulation requirements, including the processing, storage, and protection of all data. Ethical approval has been obtained from the Swedish Ethical Review Authority previously called Regional Ethical Review Board in Uppsala, reference number 2019/01170, with amendments, and reference number 2022-03146-02. The BASIC Child project was approved by the Reginal Ethical Review Board in Uppsala (Reference number 2013/423). Participants are informed about the goals of the study, and the confidentiality and security of their data will be assured. All participants provide their consent prior to each data collection time point, and they are informed that they can withdraw their participation at any time without providing a reason. All results will be continuously disseminated through international peer-reviewed journals, the project's website, social media channels, and national and international conferences.

Patient and public involvement

Patient and public involvement are essential to the current cohort profile. From pilot design to long-term follow-ups, a high degree of engagement from patients and the public continues to shape the development of this study. Our research team has also established open communication through a variety of platforms with the public from the beginning of the study. Study participants have been encouraged to contact the team regarding any comments and questions related to procedures. We have reviewed patients' and the public's feedback and we will continue to take this valuable information into account when examining our research approach and acquired data. The research team has close contact with several stakeholders, including clinical staff and patient organisations.

FINDINGS TO DATE

What have we learnt about perinatal depression correlates from the BASIC project?

The BASIC cohort project aimed to identify depression and anxiety symptom patterns and their biophysiological correlates from pregnancy to 12 months postpartum (for the full publication list: https://www.basicstudie.se/publicerade-arbeten). In this cohort, five different trajectories of perinatal depression were characterized (15). We also observed a variety of risk factors related to different trajectories of depressive symptoms, such as adverse childhood experiences, instrumental delivery, a negative delivery expectation and experience, lack of sleep, infantile colic, smoking prior to pregnancy, low partner support, lower education, unemployment, bonding difficulties, intimate partner violence, interpersonal trauma, attachment anxiety, prolonged nausea and vomiting in pregnancy, and low resilience (15, 54-61). Furthermore, we

For peer review only - http://bmjopen.bmj.com/site/about/guidelines.xhtml

demonstrated that, in healthy pregnancy, most inflammatory markers increased in the postpartum period (62). Intriguingly, several markers such as monocyte chemotactic protein 4 (MCP-4) tumour necrosis factor ligand superfamily member, hepatocyte growth factor, interleukin, fibroblast growth factor 23, and C-X-C motif chemokine 1, and STAM-Binding Protein were related to different depression trajectories (63-66). Compared to healthy women, women with antenatal depression or with SSRI treatment showed lower levels of peripheral inflammatory markers (67). When examining metabolic profiles in our cohort, there were no significant depression-specific differences in untargeted gas chromatography-mass spectrometry between women with depressive symptoms and without depressive symptoms during the antenatal phase (68). However, five metabolites (glycerol, threonine, 2-hydroxybutanoic acid, erythritol, and phenylalanine) were higher among women with postpartum depression than controls (69). Moreover, pregnancy is associated with a dysregulation of the hypothalamic-pituitary-adrenal axis and an increase in basal cortisol levels. Though the endocrine changes are prominent during pregnancy, there is no significant difference between women with depressive symptoms and without depressive symptoms (70), the difference became significant postpartum (71). Intriguingly, among women who did not have depressive symptoms during pregnancy, the levels of corticotropin-releasing hormone in the 2nd trimester were positively correlated with the levels of postpartum depressive symptoms (72). In addition, while evidence suggests that placental glucocorticoid receptors are affected by maternal depression or anxiety (73, 74), results from one of our subsets contrast with some previous reports (75, 76). However, at the gene level, the single nucleotide polymorphisms in the hydroxysteroid 11-beta dehydrogenase 2 gene, which is a key hypothalamic-pituitary-adrenal axis gene, is significantly linked to depressive symptoms in 6 weeks postpartum and the stress levels during pregnancy (77). Our data showed that women with SSRIs treatment showed higher levels of serotonin receptor 1A (HTR1A) and neuropeptide Y2 receptor (NPY2R) in the placenta compared with healthy women or women with untreated depression (78). We are now planning, using the U-BIRTH study, to analyse if the above biological differences observed in mothers perinatally exist between children with social-emotional problems and controls.

What is the impact of perinatal depression on infant's outcome?

The interplay between maternal well-being, perinatal exposure and childhood environment contribute to the cognitive, social and emotional development of the child (16, 17). The BASIC cohort underscored many important factors and potential underlying mechanisms behind perinatal depression. These findings have further laid the foundation to investigate the associations between perinatal depression and children's outcomes.

From the BASIC Child longitudinal subset, we observed that among mothers with lower levels of maternal postpartum depression, infants showed more gaze-following behaviour at 10 months (79). In another study, we observed that a mother's adverse childhood experience is highly related to depressive and anxiety symptoms throughout pregnancy and the first year postpartum. More importantly, a higher level of exposure to interpersonal traumatic events moderated the negative impact of anxiety during the 2nd trimester on an infant's attentional control from 6 to 18 months (61). In the developmental literature, gaze-following is an ability to synchronize visual attention with others (80) and this ability is linked to social learning and emotional regulation in the early years (81, 82). Attentional control in infancy has been postulated as fundamental to later learning and cognitive development (83). The negative impact of perinatal depression on infants' development was validated in our recent publication using U-BIRTH data at 18 months. We investigated the associations between different perinatal trajectories and an infant's behavioural problems at 18 months. Our results demonstrated that

For peer review only - http://bmjopen.bmj.com/site/about/guidelines.xhtml

compared to infants of healthy mothers, infants of depressed mothers showed a higher risk of behavioural problems. Maternal antenatal and persistent depression were associated with a higher degree of behavioural problems and girls were affected to a greater degree. When we analysed the mediating effect of maternal postpartum bonding, maternal persistent- and postpartum depression showed a high level of indirect effect on infants' behavioural problems, while antenatal depression had a stronger direct effect (84). Based on longitudinal data from BASIC and U-BIRTH during the first 18 months after birth, we are currently investigating how maternal depression and anxiety symptoms during pregnancy are associated with infant temperament trajectories (manuscript submitted). This investigation points at a specific importance for maternal anxiety symptoms during pregnancy that are associated with infant temperament being experienced as increasingly difficult throughout the first 18 months. This is in comparison with depressive/anhedonia symptoms that are associated with infant temperament being stably medium-high in difficulty. Also here, sex-specific effects were found with girls being more vulnerable towards maternal anxiety during pregnancy and boys towards maternal postpartum depression. Taken together, results from our subset (BASIC Child) and data from U-BIRTH support the hypothesis that there are negative influences of perinatal depression on infant's outcome at the behavioural level.

At the biological level, several studies based on the BASIC cohort report a significant impact of perinatal depressive symptoms on child biological and emotional outcomes. For instance, we looked at DNA methylation in the cord blood of new-borns, which is a form of pregenetic modification and most of the changes happen during embryogenesis (85). According to the foetal programming hypothesis (86), it is assumed that DNA methylation might be a possible underlying mechanism that contributes to cross-generational transmission. In our subset with 373 mother-infant dyads, we observed the differential DNA methylation in infants with mothers combined perinatal depressive symptoms and anxiety symptoms or mothers with SSRIs treatment compared to infants with healthy mothers (87). In our recent publication based on the U-BIRTH cohort, we used DNA extracted in cord blood at birth and examined if DNA methylation differences could affect an infant's behavioural problems at 18 months while taking perinatal depression into account. However, we didn't find evidence to support the foetal programming hypothesis (88). At the physical level, we investigated the relationship between prenatal depression, the mother's BMI, and infant birth weight. The main result showed that depressive symptoms interact with the mother's BMI. Consequently, in mothers who had a higher BMI and depressive symptoms during pregnancy, their infants were born with a greater body weight compared to infants with healthy mothers or depressed mothers with normal BMI (89). Furthermore, previous evidence has shown that skin-to-skin contact might help the infant regulate stress (90). However, mothers with perinatal depressive symptoms during pregnancy showed a delayed first breastfeeding session compared with mothers without depressive symptoms. Mothers who experienced depressive symptoms during pregnancy and had a delayed initiation of breastfeeding after birth continued to provide less exclusive breastfeeding at 6 weeks postpartum (91). Our results support the notion that both infants and depressed mothers might benefit from targeted breastfeeding support during the first hours after delivery.

STRENGTHS AND LIMITATIONS

This population-based project has one of the largest longitudinal cohorts in Scandinavian countries. This project provides comprehensive and rich data across pregnancy, infancy, childhood, and pre-teen phase. Furthermore, to disentangle the complex interaction between maternal well-being, perinatal exposure, and childhood environment and how they contribute to the cognitive, social and emotional development of the child, this study includes multidimensional data from biological, psychometrical, and behavioural measures. Not only

For peer review only - http://bmjopen.bmj.com/site/about/guidelines.xhtml

do our data extend the continuity of the previous BASIC cohort profile, but the findings from the BASIC cohort have also established the foundation for the U-BIRTH project. More specifically, results such as risk factors and inflammatory markers for perinatal depression provide us the evidence-based references to investigate the potential mechanisms of cross-generational transmission.

However, despite the relatively large sample included in the current project, the acceptance rate at the baseline from the BASIC cohort was low. Moreover, from the participants' sociodemographic information, most parents enrolled hold a degree in higher education and this has been an indication of a low-risk profile (92). This might be related to the study being conducted in a university town. In addition, mothers with depressive symptoms have a higher rate of dropout compared to mothers without depressive symptoms. Another limitation is that the cohort's representability might decrease over time due to the increased rate of missing data and decline in participation. Given these limitations, we must generalize the results with caution.

With regard to outcome measures, though assessments of child development included in the project are well accepted and widely applied in the field, most of them rely heavily on parent-report questionnaires. This could introduce information bias. Finally, our study only recruited Swedish-speaking mothers. This admittedly excludes many families with an immigrant background who might have other risk factors, such as lack of social support from extended family, language barriers, etc. This could also limit the generalizability of our results.

Collaboration

Researchers interested in collaboration may contact the corresponding author, Alkistis Skalkidou, with their request. The U-BIRTH study steering committee considers the scientific quality of the aims and methods of incoming requests in addition to the volume of the requested data or samples. The committee prioritises study questions on perinatal depression and child development.

Authors' contributions

AS conceptualized and designed of the study. EF, TKK and MR contributed significantly to the design and execution of the project. UE and HFT contribute to the continuation of the project. HFT drafted the article, and all authors revised the draft critically for intellectual content and approved the final version.

Funding statement

This project is supported by the Uppsala Region (ALF-965886 and ALF-965905), the Göran Gustafsson Foundation (Nr. 1551 A-2015), the Swedish Research Council (Nr. 523-2014-07605), Marianne och Marcus Wallenbergs Stiftelse (MMW 2011-0115), Söderström-Königska sjukhemmet (Dnr SLS-300191) to AS, and the Gillbergska Foundation (2022) to EF.

Acknowledgements

We are very grateful to all families who participated in this project, as well as to Carolina Wikström, Elisabeth Hansson and Amanda Olsson for administrative support.

Conflict of Interests Statement

All authors have no affiliations with or involvement in any organization or entity with any financial interest or non-financial interest in the subject matter or materials discussed in this manuscript.

For peer review only - http://bmjopen.bmj.com/site/about/guidelines.xhtml

References

1. O'Hara MW, Wisner KL. Perinatal mental illness: definition, description and aetiology. Best Practice & Research Clinical Obstetrics & Gynaecology. 2014;28(1):3-12.
2. Woody C, Ferrari A, Siskind D, Whiteford H, Harris M. A systematic review and meta-regression of the prevalence and incidence of perinatal depression. Journal of Affective Disorders. 2017;219:86-92.
3. Underwood L, Waldie K, D'Souza S, Peterson ER, Morton S. A review of longitudinal studies on antenatal and postnatal depression. Archives of Women's Mental Health. 2016;19(5):711-20.
4. Gavin NI, Gaynes BN, Lohr KN, Meltzer-Brody S, Gartlehner G, Swinson T. Perinatal depression: a systematic review of prevalence and incidence. Obstetrics & Gynecology. 2005;106(5 Part 1):1071-83.
5. Healey C, Morriss R, Henshaw C, Wadoo O, Sajjad A, Scholefield H, et al. Self-harm in postpartum depression and referrals to a perinatal mental health team: an audit study. Archives of Women's Mental Health. 2013;16(3):237-45.
6. Lindahl V, Pearson JL, Colpe L. Prevalence of suicidality during pregnancy and the postpartum. Archives of Women's Mental Health. 2005;8(2):77-87.
7. Li J, Yin J, Waqas A, Huang Z, Zhang H, Chen M, et al. Quality of Life in Mothers With Perinatal Depression: A Systematic Review and Meta-Analysis. Frontiers in Psychiatry. 2022;13.
8. Tirumalaraju V, Suchting R, Evans J, Goetzl L, Refuerzo J, Neumann A, et al. Risk of depression in the adolescent and adult offspring of mothers with perinatal depression: a systematic review and meta-analysis. JAMA network open. 2020;3(6):e208783-e.
9. Mwaniki MK, Atieno M, Lawn JE, Newton CR. Long-term neurodevelopmental outcomes after intrauterine and neonatal insults: a systematic review. The Lancet. 2012;379(9814):445-52.
10. Stevenson K, Lillycrop KA, Silver MJ. Fetal programming and epigenetics. Current Opinion in Endocrine and Metabolic Research. 2020;13:1-6.
11. Huot R, Brennan P, Stowe Z, Plotsky P, Walker E. Negative affect in offspring of depressed mothers is predicted by infant cortisol levels at 6 months and maternal depression during pregnancy, but not postpartum. Annals of the New York Academy of Sciences. 2004;1032(1):234-6.
12. Power J, van IJzendoorn M, Lewis AJ, Chen W, Galbally M. Maternal perinatal depression and child executive function: A systematic review and meta-analysis. Journal of Affective Disorders. 2021;291:218-34.
13. Nidey NL, Momany AM, Strathearn L, Carter KD, Wehby GL, Bao W, et al. Association between perinatal depression and risk of attention deficit hyperactivity disorder among children: A retrospective cohort study. Annals of Epidemiology. 2021;63:1-6.
14. Chen L-C, Chen M-H, Hsu J-W, Huang K-L, Bai Y-M, Chen T-J, et al. Association of parental depression with offspring attention deficit hyperactivity disorder and autism spectrum disorder: A nationwide birth cohort study. Journal of Affective Disorders. 2020;277:109-14.
15. Wikman A, Axfors C, Iliadis SI, Cox J, Fransson E, Skalkidou A. Characteristics of women with different perinatal depression trajectories. Journal of Neuroscience Research. 2020;98(7):1268-82.
16. Kingston D, Tough S, Whitfield H. Prenatal and postpartum maternal psychological distress and infant development: a systematic review. Child Psychiatry & Human Development. 2012;43(5):683-714.

For peer review only - http://bmjopen.bmj.com/site/about/guidelines.xhtml

17. Stein A, Pearson RM, Goodman SH, Rapa E, Rahman A, McCallum M, et al. Effects of perinatal mental disorders on the fetus and child. The Lancet. 2014;384(9956):1800-19.
18. Rogers A, Obst S, Teague SJ, Rossen L, Spry EA, Macdonald JA, et al. Association between maternal perinatal depression and anxiety and child and adolescent development: a meta-analysis. JAMA Pediatrics. 2020;174(11):1082-92.
19. Van Steijn DJ, Oerlemans AM, Van Aken MA, Buitelaar JK, Rommelse NN. The reciprocal relationship of ASD, ADHD, depressive symptoms and stress in parents of children with ASD and/or ADHD. Journal of Autism and Developmental Disorders. 2014;44(5):1064-76.
20. Keim SA, Daniels JL, Dole N, Herring AH, Siega-Riz AM, Scheidt PC. A prospective study of maternal anxiety, perceived stress, and depressive symptoms in relation to infant cognitive development. Early Human Development. 2011;87(5):373-80.
21. Hirschfeld RM. The comorbidity of major depression and anxiety disorders: recognition and management in primary care. Primary care companion to the Journal of Clinical Psychiatry. 2001;3(6):244.
22. Santos H, Tan X, Salomon R. Heterogeneity in perinatal depression: how far have we come? A systematic review. Archives of Women's Mental Health. 2017;20(1):11-23.
23. Axfors C, Bränn E, Henriksson HE, Hellgren C, Kallak TK, Fransson E, et al. Cohort profile: the Biology, Affect, Stress, Imaging and Cognition (BASIC) study on perinatal depression in a population-based Swedish cohort. BMJ open. 2019;9(10):e031514.
24. Cox JL, Holden JM, Sagovsky R. Detection of postnatal depression: development of the 10-item Edinburgh Postnatal Depression Scale. The British Journal of Psychiatry. 1987;150(6):782-6.
25. Zung WW. A self-rating depression scale. Archives of General psychiatry. 1965;12(1):63-70.
26. Beck AT, Epstein N, Brown G, Steer RA. An inventory for measuring clinical anxiety: psychometric properties. Journal of Consulting and Clinical Psychology. 1988;56(6):893.
27. Beck AT, Steer RA, Carbin MG. Psychometric properties of the Beck Depression Inventory: Twenty-five years of evaluation. Clinical Psychology Review. 1988;8(1):77-100.
28. Speilberger CD, Gorsuch R, Lushene R, Vagg P, Jacobs G. Manual for the state-trait anxiety inventory. Palo Alto, CA: Consulting Psychologists. 1983.
29. Horowitz M, Wilner N, Alvarez W. Impact of Event Scale: A measure of subjective stress. Psychosomatic Medicine. 1979;41(3):209-18.
30. Frans Ö, Rimmö PA, Åberg L, Fredrikson M. Trauma exposure and post-traumatic stress disorder in the general population. Acta Psychiatrica Scandinavica. 2005;111(4):291-0.
31. Feeney J, Noller P, Hanrahan M. Assessing adult attachment. In: Sperling M, Berman W, editors. Attachment in adults: clinical and developmental perspectives. New York: Guilford Press; 1994. p. 128-52.
32. Greenwald R, Rubin A. Assessment of posttraumatic symptoms in children: Development and preliminary validation of parent and child scales. Research on Social Work Practice. 1999;9(1):61-75.
33. Larsson I. LITE-P, life incidence of traumatic events. Translation into Swedish, with permission from the author In: R Greenwald. 2003.
34. Brockington IF, Fraser C, Wilson D. The postpartum bonding questionnaire: a validation. Archives of Women's Mental Health. 2006;9(5):233-42.
35. Brockington IF, Oates J, George S, Turner D, Vostanis P, Sullivan M, et al. A screening questionnaire for mother-infant bonding disorders. Archives of Women's Mental Health. 2001;3(4):133-40.

For peer review only - http://bmjopen.bmj.com/site/about/guidelines.xhtml

36. Antonovsky A. The structure and properties of the sense of coherence scale. Social Science & Medicine. 1993;36(6):725-33.
37. Gustavsson JP, Bergman H, Edman G, Ekselius L, Von Knorring L, Linder J. Swedish universities Scales of Personality (SSP): construction, internal consistency and normative data. Acta Psychiatrica Scandinavica. 2000;102(3):217-25.
38. Boyce P, Hickey A, Gilchrist J, Talley N. The development of a brief personality scale to measure vulnerability to postnatal depression. Archives of Women's Mental Health. 2001;3(4):147-53.
39. Wagnild GM, Young HM. Development and psychometric evaluation of the resilience scale. Journal of Nursing Measurement. 1993;1(2):165-17847.
40. Spitzer RL, Kroenke K, Williams JB, Group PHQPCS, Group PHQPCS. Validation and utility of a self-report version of PRIME-MD: the PHQ primary care study. JAMA. 1999;282(18):1737-44.
41. Maruish ME. User's manual for the SF-36v2 Health Survey: Quality Metric Incorporated; 2011.
42. Group TE. EuroQol-a new facility for the measurement of health-related quality of life. Health Policy. 1990;16(3):199-208.
43. Dumas JE, Nissley J, Nordstrom A, Smith EP, Prinz RJ, Levine DW. Home chaos: Sociodemographic, parenting, interactional, and child correlates. Journal of Clinical Child and Adolescent Psychology. 2005;34(1):93-104.
44. Haskett ME, Ahern LS, Ward CS, Allaire JC. Factor structure and validity of the parenting stress index-short form. Journal of Clinical Child & Adolescent Psychology. 2006;35(2):302-12.
45. Östberg M, Hagekull B. Frågeformulär angående föräldrapåfrestning (The Swedish Parenthood Stress Questionnaire-SPSQ) Beskrivning av instrumentets konstruktion, normdata, reliabilitet och validitet. Opublicerat manuskript, Uppsala Universitet, Sverige; 2012.
46. Rothbart MK. Measurement of temperament in infancy. Child Development. 1981:569-78.
47. Putnam SP, Gartstein MA, Rothbart MK. Measurement of fine-grained aspects of toddler temperament: The Early Childhood Behavior Questionnaire. Infant Behavior and Development. 2006;29(3):386-401.
48. Achenbach TM, Rescorla LA. Manual for the ASEBA preschool forms and profiles: Burlington, VT: University of Vermont, Research center for children, youth …; 2000.
49. Achenbach TM. Manual for ASEBA school-age forms & profiles. University of Vermont, Research Center for Children, Youth & Families. 2001.
50. Rescorla L, Alley A. Validation of the language development survey (LDS). 2001.
51. Kadesjö B, Janols L-O, Korkman M, Mickelsson K, Strand G, Trillingsgaard A, et al. The FTF (Five to Fifteen): the development of a parent questionnaire for the assessment of ADHD and comorbid conditions. European Child & Adolescent Psychiatry. 2004;13(3):iii3-iii13.
52. Korkman M, Kadesjö B, Trillingsgaard A, Mickelsson K, Janols L-O, Strand G, et al. Frågeformulär för utredning av utveckling och beteende hos 5 till 15 år gamla barn. 2005.
53. Gredebäck G, Forssman L, Lindskog M, Kenward B. The BasicChild Project. 2019.
54. Axfors C, Sylvén S, Ramklint M, Skalkidou A. Adult attachment's unique contribution in the prediction of postpartum depressive symptoms, beyond personality traits. Journal of Affective Disorders. 2017;222:177-84.
55. Iliadis SI, Axfors C, Johansson S, Skalkidou A, Mulic-Lutvica A. Women with prolonged nausea in pregnancy have increased risk for depressive symptoms postpartum. Scientific Reports. 2018;8(1):1-9.

For peer review only - http://bmjopen.bmj.com/site/about/guidelines.xhtml

56. Asif S, Mulic-Lutvica A, Axfors C, Eckerdal P, Iliadis SI, Fransson E, et al. Severe obstetric lacerations associated with postpartum depression among women with low resilience–a Swedish birth cohort study. BJOG: An International Journal of Obstetrics & Gynaecology. 2020;127(11):1382-90.
57. Eckerdal P, Georgakis MK, Kollia N, Wikström AK, Högberg U, Skalkidou A. Delineating the association between mode of delivery and postpartum depression symptoms: a longitudinal study. Acta obstetricia et gynecologica Scandinavica. 2018;97(3):301-11.
58. Eckerdal P, Kollia N, Löfblad J, Hellgren C, Karlsson L, Högberg U, et al. Delineating the association between heavy postpartum haemorrhage and postpartum depression. PloS one. 2016;11(1):e0144274.
59. Henriksson HE, Sylvén SM, Kallak TK, Papadopoulos F, Skalkidou A. Seasonal patterns in self-reported peripartum depressive symptoms. European Psychiatry. 2017;43:99-108.
60. Kimmel MC, Fransson E, Cunningham JL, Brann E, Grewen K, Boschiero D, et al. Heart rate variability in late pregnancy: exploration of distinctive patterns in relation to maternal mental health. Translational Psychiatry. 2021;11(1):1-11.
61. Tu H-F, Skalkidou A, Lindskog M, Gredebäck G. Maternal childhood trauma and perinatal distress are related to infants' focused attention from 6 to 18 months. Scientific Reports. 2021;11(1):24190.
62. Bränn E, Edvinsson Å, Rostedt Punga A, Sundström-Poromaa I, Skalkidou A. Inflammatory and anti-inflammatory markers in plasma: from late pregnancy to early postpartum. Scientific Reports. 2019;9(1):1-10.
63. Osimo EF, Pillinger T, Rodriguez IM, Khandaker GM, Pariante CM, Howes OD. Inflammatory markers in depression: a meta-analysis of mean differences and variability in 5,166 patients and 5,083 controls. Brain, Behavior, and Immunity. 2020;87:901-9.
64. Henriksson HE, White RA, Iliadis SI, Fransson E, Papadopoulos FC, Sundström-Poromaa I, et al. Spring peaks and autumn troughs identified in peripheral inflammatory markers during the peripartum period. Scientific Reports. 2019;9(1):1-13.
65. Bränn E, Fransson E, White RA, Papadopoulos FC, Edvinsson Å, Kamali-Moghaddam M, et al. Inflammatory markers in women with postpartum depressive symptoms. Journal of Neuroscience Research. 2020;98(7):1309-21.
66. Bränn E, Papadopoulos F, Fransson E, White R, Edvinsson Å, Hellgren C, et al. Inflammatory markers in late pregnancy in association with postpartum depression—a nested case-control study. Psychoneuroendocrinology. 2017;79:146-59.
67. Edvinsson Å, Bränn E, Hellgren C, Freyhult E, White R, Kamali-Moghaddam M, et al. Lower inflammatory markers in women with antenatal depression brings the M1/M2 balance into focus from a new direction. Psychoneuroendocrinology. 2017;80:15-25.
68. Henriksson HE, Malavaki C, Bränn E, Drainas V, Lager S, Iliadis SI, et al. Blood plasma metabolic profiling of pregnant women with antenatal depressive symptoms. Translational Psychiatry. 2019;9(1):1-9.
69. Bränn E, Malavaki C, Fransson E, Ioannidi M-K, Henriksson HE, Papadopoulos FC, et al. Metabolic Profiling Indicates Diversity in the Metabolic Physiologies Associated With Maternal Postpartum Depressive Symptoms. Frontiers in Psychiatry. 2021;12.
70. Hellgren C, Åkerud H, Skalkidou A, Sundström-Poromaa I. Cortisol awakening response in late pregnancy in women with previous or ongoing depression. Psychoneuroendocrinology. 2013;38(12):3150-4.
71. Iliadis SI, Comasco E, Sylvén S, Hellgren C, Sundström Poromaa I, Skalkidou A. Prenatal and postpartum evening salivary cortisol levels in association with peripartum depressive symptoms. PloS one. 2015;10(8):e0135471.

For peer review only - http://bmjopen.bmj.com/site/about/guidelines.xhtml

72. Iliadis SI, Sylvén S, Hellgren C, Olivier JD, Schijven D, Comasco E, et al. Mid-pregnancy corticotropin-releasing hormone levels in association with postpartum depressive symptoms. Depression and Anxiety. 2016;33(11):1023-30.
73. Togher KL, Treacy E, O'Keeffe GW, Kenny LC. Maternal distress in late pregnancy alters obstetric outcomes and the expression of genes important for placental glucocorticoid signalling. Psychiatry Research. 2017;255:17-26.
74. Reynolds R, Pesonen A-K, O'reilly J, Tuovinen S, Lahti M, Kajantie E, et al. Maternal depressive symptoms throughout pregnancy are associated with increased placental glucocorticoid sensitivity. Psychological Medicine. 2015;45(10):2023-30.
75. Edvinsson Å, Hoyer A, Hansson M, Kallak TK, Sundström-Poromaa I, Skalkidou A, et al. Placental glucocorticoid receptors are not affected by maternal depression or SSRI treatment. Upsala Journal of Medical Sciences. 2020;125(1):30-6.
76. Hellgren C, Comasco E, Skalkidou A, Sundström-Poromaa I. Allopregnanolone levels and depressive symptoms during pregnancy in relation to single nucleotide polymorphisms in the allopregnanolone synthesis pathway. Hormones and Behavior. 2017;94:106-13.
77. Skalkidou A, Poromaa IS, Iliadis SI, Huizink AC, Hellgren C, Freyhult E, et al. Stress-related genetic polymorphisms in association with peripartum depression symptoms and stress hormones: a longitudinal population-based study. Psychoneuroendocrinology. 2019;103:296-305.
78. Edvinsson Å, Hellgren C, Kunovac Kallak T, Åkerud H, Skalkidou A, Stener-Victorin E, et al. The effect of antenatal depression and antidepressant treatment on placental tissue: a protein-validated gene expression study. BMC Pregnancy and Childbirth. 2019;19(1):1-13.
79. Astor K, Lindskog M, Forssman L, Kenward B, Fransson M, Skalkidou A, et al. Social and emotional contexts predict the development of gaze following in early infancy. Royal Society Open Science. 2020;7(9):201178.
80. Driver IV J, Davis G, Ricciardelli P, Kidd P, Maxwell E, Baron-Cohen S. Gaze perception triggers reflexive visuospatial orienting. Visual Cognition. 1999;6(5):509-40.
81. Brooks R, Meltzoff AN. Connecting the dots from infancy to childhood: A longitudinal study connecting gaze following, language, and explicit theory of mind. Journal of Experimental Child Psychology. 2015;130:67-78.
82. Mundy P. Annotation: The neural basis of social impairments in autism: The role of the dorsal medial-frontal cortex and anterior cingulate system. Journal of Child Psychology and Psychiatry. 2003;44(6):793-809.
83. Posner MI, Rothbart MK, Sheese BE, Voelker P. Developing attention: behavioral and brain mechanisms. Advances in Neuroscience. 2014;2014.
84. Fransson E, Sörensen F, Kallak TK, Ramklint M, Eckerdal P, Heimgärtner M, et al. Maternal perinatal depressive symptoms trajectories and impact on toddler behavior–the importance of symptom duration and maternal bonding. Journal of Affective Disorders. 2020;273:542-51.
85. Reik W. Stability and flexibility of epigenetic gene regulation in mammalian development. Nature. 2007;447(7143):425-32.
86. Barker DJP. The developmental origins of adult disease. Journal of the American College of Nutrition. 2004;23(sup6):588S-95S.
87. Kallak TK, Bränn E, Fransson E, Johansson Å, Lager S, Comasco E, et al. DNA methylation in cord blood in association with prenatal depressive symptoms. Clinical Epigenetics. 2021;13(1):1-14.

For peer review only

88. Kallak TK, Fransson E, Bränn E, Berglund H, Lager S, Comasco E, et al. Maternal prenatal depressive symptoms and toddler behavior: an umbilical cord blood epigenome-wide association study. Translational Psychiatry. 2022;12(1):1-11.
89. Petursdottir Maack H, Skalkidou A, Sjöholm A, Eurenius-Orre K, Mulic-Lutvica A, Wikström A-K, et al. Maternal body mass index moderates antenatal depression effects on infant birthweight. Scientific Reports. 2019;9(1):1-7.
90. Ionio C, Ciuffo G, Landoni M. Parent–infant skin-to-skin contact and stress regulation: A systematic review of the literature. International Journal of Environmental Research and Public Health. 2021;18(9):4695.
91. Cato K, Sylvén SM, Georgakis MK, Kollia N, Rubertsson C, Skalkidou A. Antenatal depressive symptoms and early initiation of breastfeeding in association with exclusive breastfeeding six weeks postpartum: a longitudinal population-based study. BMC Pregnancy and Childbirth. 2019;19(1):1-11.
92. Lancaster CA, Gold KJ, Flynn HA, Yoo H, Marcus SM, Davis MM. Risk factors for depressive symptoms during pregnancy: a systematic review. American Journal of Obstetrics and Gynecology. 2010;202(1):5-14.

For peer review only

For peer review only - http://bmjopen.bmj.com/site/about/guidelines.xhtml

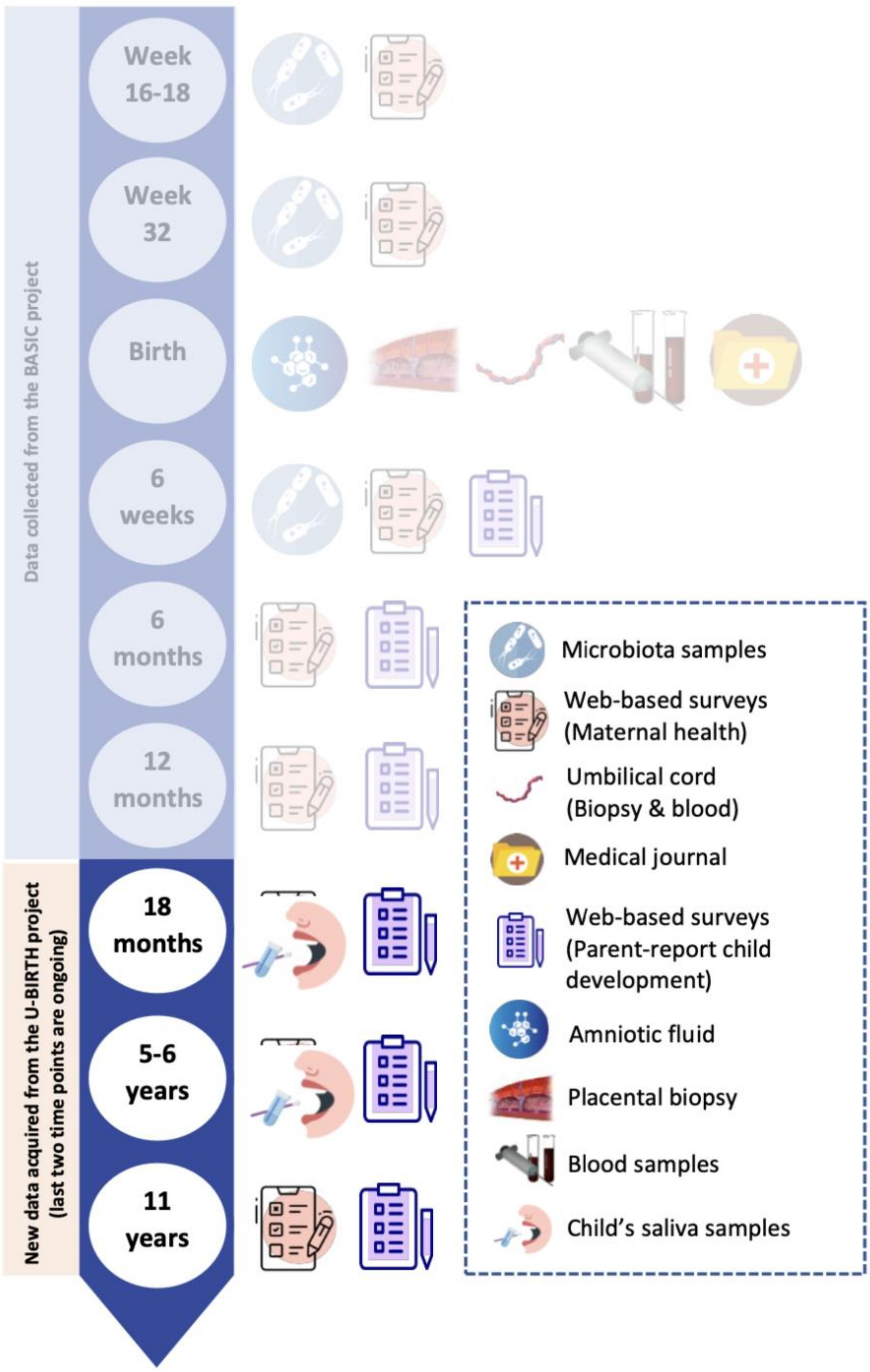

Figure 1. Data timeline included in the U-BIRTH cohort

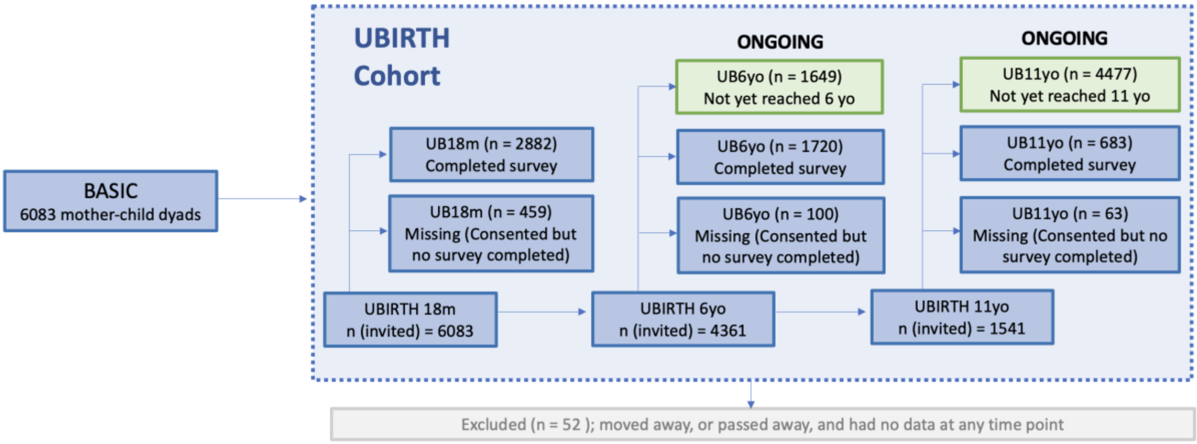

Figure 2. Flowchart of the U-BIRTH study participants (UB: U-BIRTH)

BMJ Open

Cohort profile: the U-BIRTH study on peripartum depression and child development in Sweden

Journal:	*BMJ Open*
Manuscript ID	bmjopen-2023-072839.R1
Article Type:	Cohort profile
Date Submitted by the Author:	05-Sep-2023
Complete List of Authors:	Tu, Hsing-Fen; Uppsala University, Department of Women's and Children's Health; Uppsala University, Department of Psychology<br>Fransson, Emma; Uppsala University, Department of Women's and Children's Health; Karolinska Institutet, Centre for Translational Microbiome Research, Department of Microbiology, Tumor and Cell Biology<br>Kunovac Kallak, Theodora; Uppsala University, Department of Women's and Children's Health<br>Elofsson, Ulf; Uppsala University, Department of Women's and Children's Health<br>Ramklint, Mia; Uppsala University, Department of Neuroscience, Psychiatry<br>Skalkidou, Alkistis; Uppsala University, Department of Women's and Children's Health
**Primary Subject Heading**:	Mental health
Secondary Subject Heading:	Epidemiology
Keywords:	GYNAECOLOGY, PSYCHIATRY, Community child health < PAEDIATRICS, Depression & mood disorders < PSYCHIATRY

SCHOLARONE™
Manuscripts

I, the Submitting Author has the right to grant and does grant on behalf of all authors of the Work (as defined in the below author licence), an exclusive licence and/or a non-exclusive licence for contributions from authors who are: i) UK Crown employees; ii) where BMJ has agreed a CC-BY licence shall apply, and/or iii) in accordance with the terms applicable for US Federal Government officers or employees acting as part of their official duties; on a worldwide, perpetual, irrevocable, royalty-free basis to BMJ Publishing Group Ltd ("BMJ") its licensees and where the relevant Journal is co-owned by BMJ to the co-owners of the Journal, to publish the Work in this journal and any other BMJ products and to exploit all rights, as set out in our licence.

The Submitting Author accepts and understands that any supply made under these terms is made by BMJ to the Submitting Author unless you are acting as an employee on behalf of your employer or a postgraduate student of an affiliated institution which is paying any applicable article publishing charge ("APC") for Open Access articles. Where the Submitting Author wishes to make the Work available on an Open Access basis (and intends to pay the relevant APC), the terms of reuse of such Open Access shall be governed by a Creative Commons licence – details of these licences and which Creative Commons licence will apply to this Work are set out in our licence referred to above.

Other than as permitted in any relevant BMJ Author's Self Archiving Policies, I confirm this Work has not been accepted for publication elsewhere, is not being considered for publication elsewhere and does not duplicate material already published. I confirm all authors consent to publication of this Work and authorise the granting of this licence.

For peer review only

Cohort profile: the U-BIRTH study on peripartum depression and child development in Sweden

Hsing-Fen Tu[1,2], Emma Fransson[1,3], Theodora Kunovac Kallak[1], Ulf Elofsson[1], Mia Ramklint[4], Alkistis Skalkidou[1]*

Affiliations

[1]Department of Women's and Children's Health, Uppsala University, Uppsala, Sweden

[2]Department of Psychology, Uppsala University, Uppsala, Sweden

[3]Centre for Translational Microbiome Research, Department of Microbiology, Tumor and Cell Biology, Karolinska Institutet, Stockholm, Sweden

[4]Department of Neuroscience, Psychiatry, Uppsala University, Uppsala, Sweden

*Corresponding author

Email: alkistis.skalkidou@kbh.uu.se

ABSTRACT

Purpose

The current U-BIRTH cohort extends our previous cohort Biology, Affect, Stress, Imaging and Cognition (BASIC), assessing the development of children up to 11 years after birth. The U-BIRTH study aims to (1) assess the impact of exposure to peripartum mental illness on the children´s development taking into account biological and environmental factors during intrauterine life and childhood; (2) identify early predictors of child neurodevelopmental and psychological problems using biophysiological, psychosocial, and environmental variables available during pregnancy and early postpartum.

For peer review only - http://bmjopen.bmj.com/site/about/guidelines.xhtml

Participants

All mothers participating in the previous BASIC cohort are invited, and mother-child dyads recruited in the U-BIRTH study are consecutively invited to questionnaire assessments and biological sampling when the child is 18 months, 6 years, and 11 years old. Data collection at 18 months (n = 2882) has been completed. Consent for participation has been obtained from 1946 families of children having reached age 6 and from 698 families of children having reached age 11 years.

Findings to date

Based on the complete data from pregnancy to 18 months postpartum, peripartum mental health was significantly associated with the development of attentional control and gaze-following behaviours, which are critical to cognitive and social learning later in life. Moreover, infants of depressed mothers had an elevated risk of difficult temperament and behavioural problems compared to infants of non-depressed mothers. Analyses of biological samples showed that peripartum depression and anxiety were related to DNA methylation differences in infants. However, there were no methylation differences in relation to infants' behavioural problems at 18 months of age.

Future plans

Given that the data collection at 18 months is complete, analyses are now being undertaken. Currently, assessments for children reaching 6 and 11 years are ongoing.

Strengths and limitations

For peer review only

For peer review only - http://bmjopen.bmj.com/site/about/guidelines.xhtml

- This population-based study provides rich data across pregnancy, infancy, early childhood, and the pre-teen periods; the sample corresponds to one of the largest cohorts within the Scandinavian countries.
- To disentangle how the complex interactions between maternal well-being and health, peripartum exposures, and childhood environment contribute to the cognitive, social and emotional development of the child, this study includes data from biological, psychosocial, and behavioural assessments using a longitudinal design.
- The cohort's representability is relatively low at about 10% of the background population, impacting on the generalizability of the findings.
- Though the assessments of child development included in the project are well accepted and widely applied in the field, most of them rely mainly on parent-report questionnaires, which could introduce information bias.

INTRODUCTION

Peripartum depression, a significant complication that affects many women during pregnancy and/or the first year postpartum (1), affects one in 4-7 pregnancies (2-5) and poses a serious global public health concern. Peripartum depression heightens the risk of maternal self-harm and suicidal acts (6, 7) and has a substantial and long-term impact during the most vulnerable window of opportunity for the human brain development (8). Exposure to peripartum depression during this critical period might result in lasting physical, social-emotional, neurological, and cognitive problems in children (9-13), even into adulthood (14). However, while the long-term associations between peripartum mental health and child outcomes are well-documented (15), the underlying mechanisms remain less understood. Given the high degree of complexity and the non-linear association between the trajectories of maternal

For peer review only - http://bmjopen.bmj.com/site/about/guidelines.xhtml

depression and children's development (16, 17), it is challenging to study factors deeply embedded in the temporal, social, cultural, biological, genetic, and physical environment, etc. The U-BIRTH cohort, builds on the BASIC (Biology, Affect, Stress, Imaging and Cognition) study (18), aiming to provide an in-depth understanding of the relationships between types of peripartum depression and the developmental trajectories of children, as well as the possible mechanisms of cross-generational transmission. The BASIC study explored various maternal factors linked to peripartum depression, enhancing our understanding of the mechanisms. The combination with BASIC study and the U-BIRTH cohort study will aid in identifying individual risk profiles and support personalised early prevention and intervention programs.

Peripartum depression affects both mothers and children daily life and elevates the risk of the children for poor self-regulation (10), delayed executive functions (11), and neurodevelopmental disorders, such as attention deficit hyperactivity disorder and autism spectrum disorder (12, 13). Meanwhile, mothers with peripartum depression might have a higher risk for chronic maternal depression (16). Children of depressed mothers are at risk of various problems in face challenges in social, cognitive, and academic functioning compared to those of non-depressed mothers (15, 19-21). To capture the full path of the cross-generational transmission of child development, the identification of early risk factors for peripartum depressive symptoms has been an important topic in the field. Previous research has shown potential factors like inflammatory biomarkers (e.g. increased levels of IL-6, IL-8, CRP, and TNF-α, see review by Silva-Fernandes et al., 22), genetic factors (e.g. 5-HTT, catechol-O-methyl-transferase, mono-amine-oxidase type A, etc.) (23), and psychosocial stressors (e.g. high life stress, lack of social support, history of depression, etc.) for peripartum depression (24-28). It is essential to further elucidate the longitudinal and dynamic relationship between these potential risk factors, maternal depressive symptoms, and child development.

For peer review only - http://bmjopen.bmj.com/site/about/guidelines.xhtml

The U-BIRTH study, drawing on life course epidemiology concepts and the significance of early life factors in long-term health (29), sets out to study the impact of maternal depressive trajectories on children´s social and emotional development. In summary, the U-BIRTH study thus aims to (i) assess the impact of exposure to peripartum mental illness on children´s development taking into account biological, psychological, and environmental factors during intrauterine life and childhood; (ii) identify early predictors of child neurodevelopmental and psychological problems using biophysiological, psychosocial, and environmental variables available during pregnancy and early postpartum. Specific objectives of the current protocol include: (i) presenting a detailed description of the U-BIRTH study, a continuation of the BASIC cohort with follow-ups up to 11 years after delivery, and (ii) giving updated summaries of the previous findings of associations between peripartum mental health and offspring's outcomes from both the BASIC and U-BIRTH studies. Ultimately, we hope to identify predictors as well as risk- and protective factors that might support future prevention and intervention programs.

COHORT DESCRIPTION

Study design

The U-BIRTH study, an extension of the BASIC study (18) is hosted at the Department of Obstetrics and Gynaecology at Uppsala University Hospital. The BASIC study (from September 2009 to November 2018), involved 5492 women (in 6478 pregnancies, ca. 22% of all Uppsala County births). During the BASIC study recruitment, all Swedish-speaking women aged 18 years and older with a routine ultrasound at Uppsala University Hospital were invited to participate. Exclusion criteria included age under 18 years, inadequate Swedish proficiency, protected identities, bloodborne infections and/or non-viable pregnancy detected through

For peer review only - http://bmjopen.bmj.com/site/about/guidelines.xhtml

routine ultrasound. These criteria remain the same in the U-BIRTH study. Women received written information by post and provided their written consent for modalities they wished to take part in (e.g. questionnaires, blood samples, tissue samples, or microbiota samples). Participating women were followed up from gestational week 16–18 through 12 months postpartum. In the BASIC study (18), data were collected using various psychometric self-report instruments and biological samples (more information: https://www.basicstudie.se/). For the U-BIRTH study (https://www.ubirth.se/), initiated in 2012, all the BASIC study participants not opting out in any way were (will be) invited when their children reached (or reach) 18 months, 6 and 11 years of age. The youngest participants were born in April 2019. Data acquisition for 6-year-olds and 11-year-olds is expected to finish in the summer 2025 and 2030. Prior to each time point for assessments, consent from all participating mother-child dyads is (will be) obtained. Many participating mother-child dyads already have data from multiple time points acquired within the BASIC study, with the sociodemographic information available in Table 1. In the U-BIRTH study, all recruited mother-child dyads are invited to a series of assessments when the child is at age of 18 months, 6 years, and 11 years (Table 2). Figure 1 illustrates the data timeline included in the protocol. For a full list of assessment tools for mothers and children in both BASIC and U-BIRTH studies, please see Supplementary Table 1. Currently, 3525 families have completed at least one assessment and 52 mother-child dyads dropped out due to external factors (e.g. death of the mother or the child or the family moved away). Overall, all children have reached 18 months of age and the data acquisition of this phase is completed. There were 6083 invitations to the study for the 18 months postpartum, and 3373 mother-infant dyads (55%) consented to participate. Among those consenting to participate in the survey, 85% completed the survey at 18 months postpartum. Seventy-three percent of the children have today reached 6 years of age. There were 4340 invitations sent, and 1409 (32%) mother-child dyads have fully completed the assessments. Moreover, 1547

For peer review only - http://bmjopen.bmj.com/site/about/guidelines.xhtml

For peer review only

(25%) children have reached 11 years of age and 624 have completed the assessments. Families not responding to the invitation receive one email reminder. Families who agree to participate but do not answer the survey receive three email reminders. The flowchart for participants is presented in Figure 2. For those who decided to drop out without any external cause (e.g. death of the child or the mother), some provide reasons such as moving away, while others did not specify. For non-respondents, we send a reminder but cannot ascertain the reasons.

For peer review only

For peer review only - http://bmjopen.bmj.com/site/about/guidelines.xhtml

Table 1. Sociodemographic information of the U-BIRTH cohort and the general population of Uppsala County and Sweden

	U-BIRTH (n = 3525*)			Uppsala County† (2009–2016, n = 32 258)	Sweden† (2009–2016, n = 902698)
**Variable**	**Available data (n)**	**% or mean**	**Missing, n**	**% or mean**	**% or mean**
Maternal age (mean, sd)	3374	31.8 (±4.4)	151	30.4 (±5.2)	30.3 (±5.3)
Country of origin					
Scandinavia	3069	87.1	227	80.3	76.6
Other countries	229	6.5		19.7	23.3
Education					
≤ 12 years	520	17	471	46.7	48.5
Cohabiting with the 2nd legal guardian	2790	97.2	656	95.7	93.1
BMI before pregnancy kg/m^2	3094	23.7 (±4.2)	431		
< 18.5	93	2.6		2.2	2.9
18.5 ≤ BMI < 25	2146	60.9		58.6	65.6
25 ≤ BMI < 30	594	16.9		25.9	25.3
≥ 30	261	7.4		13.4	13.0
Infant birth weight, gram	3257	3593 (±546.6)	268	3498	3504
Infant birth weight < 2500g	92	2.8 %		5.1	4.4
Infant birth weight ≥ 4500g	142	4.4		3.9	3.5
Primiparous	1547	47.5	265	43.8	43.9
Premature delivery (< week 37)	168	5.2	265	7.4	6.0
Apgar 5 < 7	40	1.2	282	2.0	1.3

BMI: Body mass index. Percentages are given in relation to available data from women.
*Data downloaded on 2023-01-27. At this time points, there were 3525 families already completed at least one time assessment. These children were all born between January 2010 and April 2019. †Data retrieved on 2023-01-27 from the Swedish Medical Birth Register and Swedish National board of Health and Welfare (www.socialstyrelsen.se).

For peer review only

For peer review only - http://bmjopen.bmj.com/site/about/guidelines.xhtml

Insert Figure 1 about here

Figure 1. U-BIRTH study timeline

Insert Figure 2 about here

Figure 2. Flowchart of the U-BIRTH study participants.

For peer review only - http://bmjopen.bmj.com/site/about/guidelines.xhtml

Table 2. Content of child outcome assessments and maternal surveys included in the U-BIRTH study

Age of the child	18 months	6 years	11 years
**Domain of measure**			
**Child outcome assessments**			
General measure	Child's growth, traits, and behavior*; general Screening†	Child's growth, traits, and behavior*; general Screening†	Child's growth, traits, and behavior*; general Screening†
Sleep	Sleeping habits and problems*		
Social-emotional development	Relationship with parents*	Child's social skills*	Child's social skills and relationship*
Behavior	CBCL (1.5); ECBQ	CBCL (1.5-5)	CBCL (6-18)
Language development	CBCL-LDS<br>General Screening†	Child's language development*<br>General Screening†	Child's language health*
Neuropsychological development		FTF	FTF
School	Participation in preschool (parent-report)*	School performance (parent-report)*	Child's school performance (parent-report* and register data††)
Biological sample	Saliva sample	Saliva sample	
Others	Free time activities*	Child's chores*; child's free-time*	Child's chores*; child's free-time*
**Maternal surveys**			
Depressive symptoms	DSRS	PHQ-9	PHQ-9; DSM-5 Self-Rated Level 1 CCSM
Trauma and stressful events	SLE; SPSQ	New SLE	New SLE
Medical information	Current medication*<br>Medical journal	Current medication*<br>Medical journal	Current medication*<br>Medical journal
Lifestyles	Alcohol*; smoking*; tobacco*	Alcohol*; smoking*; tobacco*	Alcohol*; smoking*; tobacco*
Sleep	Sleep quality*	Sleep quality*	Sleep quality*
Others	CHAOS; chores and activity*; life right now*; PSS; SF-36; second parent's status*; social life*	Another pregnancy*; CHAOS; chores and activities*; life right now*; maternal leave*; partner's status*; previous breastfeeding*; PSI; SES*; SF-36; social life*	CHAOS; EQ-5; PSQ; SOC; SES*

CBCL: Child Behavior Checklist; CBCL-LDS: Child Behavior Checklist, Language Development Survey; CHAOS: Confusion, Hubbub and Order Scale ;DSM-5: the Diagnostic and Statistical Manual of Mental Disorders, the 5th Edition; DSRS: Depression Self-Rating Scale; ECBQ: Early Childhood Behavior Questionnaire; EQ-5D: Health-Related Quality of Life Questionnaire; FTF: Five to Fifteen; PHQ9: Patient Health Questionnaire; PSI: Parenting Stress Index; PSQ: Parenting Style Questionnaire; PSS: Perceived Stress Scale; SES: socioeconomic status; SF-36: The 36-Item Short Form Health Survey; SLE: Stressful Life Events Scale; SOC-29: Sense of Coherence; SPSQ: Swedish Parenthood Stress Questionnaire. Period of usage in the BASIC is stated in parentheses if other than 2009–2017. *Questions designed by the research team. †Data source: the National Board of Health and Welfare https://www.socialstyrelsen.se/. Information includes the records related to referral and visit to a speech therapist at age of 2, 3, 4, and 5 years. †† Data source: the Swedish National Agency for Education (skolverket) https://www.skolverket.se/skolutveckling/statistik.
For a full list of assessments from pregnancy to 11 years after birth, please see Supplementary Table 1.

For peer review only

Data collection

In the U-BIRTH, three follow-up timepoints include mother-reported instruments and saliva sampling from children at 18 months and 6 years of age. For detailed measures and timeline, please see Tables 2 and Figures 1, and 2 (a full list covers information in both BASIC and U-BIRTH, see Supplementary Table 1). Mothers are also invited to complete the Patient Health Questionnaire, PHQ-9 at ages of 6 and 11 years of their children. Furthermore, mothers are administered the DSM-5-TR Self-Rated Level 1 Cross-Cutting Symptom Measure (30) when their children reach 11 years of age. Other repeated maternal measures include lifestyles (e.g. Body Mass Index, diet, consumption of alcohol and tobacco), sleep habits, social environment, quality of life (SF-36 (31)), health-related quality of life, EQ-5D (32), home environment (the Confusion, Hubbub, and Order Scale, CHAOS (33)), parenting experiences (the Parenting Stress Index short form, PSI (34); the Swedish Parenthood Stress Questionnaire, SPSQ, (35), and general living situation details, etc. Regarding biological data, as illustrated in Figure 1, the BASIC study collected maternal blood samples, microbiota samples, amniotic fluid, umbilical cord biopsy, and foetal blood samples at birth. For the description of biological data collection, handling, and storage, please see the BASIC cohort profile (18).

Children's outcome measures include general physical measures, sleep behaviour, social-emotional development, behaviour, language development, neurodevelopment, school participation and performance, and more (e.g. diet and free time activities, etc.; Table 2). General physical measures include growth data collected at birth, 18 months, 6 years, and 11 years. Sleep behaviours measured at 18 months of age focus on a child's sleep habits or problems. Evaluations of the relationship with parents are scheduled at the age of 18 months, 6 and 11 years. Children's social skills are measured at 6 and 11 years old. Parent-reported behavioural measures are obtained at 18 months (the Early Childhood Behaviour Questionnaire,

For peer review only - http://bmjopen.bmj.com/site/about/guidelines.xhtml

ECBQ (36), the Child Behaviour Checklist for 1.5–five years old (37)); and 6 years (the Child Behaviour Checklist for 1.5–5 years old (37)) and 11 years of age (the Child Behaviour Checklist for 6–18 years old (38)). Language development is measured at 18 months, 6 years (the Language Development Survey (39)) , and 11 years of age. The Five to Fifteen questionnaire, FTF (40, 41), is applied to identify symptoms or problems related to attention deficit hyperactivity disorder at the age of 6 and 11 years. The survey also covers daily life, including school participation, household activities, and free time activities, at 18 months, 6, and 11 years of age. Additionally, with parental consent to link data to national register, we collect information on maternal diagnoses, children's diagnoses, child growth, medical referrals, breastfeeding information during the first years postpartum for further analysis. Importantly, with parents' consent to access the information from the medical journals and register data, we are able to identify children at the age of 11 years with diagnoses (e.g. attention deficit/hyperactivity disorder or autistic spectrum disorder) and potentially establish predictive models. Moreover, a subset of the participants was assessed from pregnancy to 30 months postpartum in collaboration with the Uppsala Child and Baby Lab at the Department of Psychology at Uppsala University (the BASIC Child Project, for the project descriptions, https://nyu.databrary.org/volume/828, 42) between 2014 and 2018. Infants' cognitive and social-emotional development was assessed using behavioural observations, eye-tracking measures, and parent-reports. Inhibitory control, executive functions, working memories, and parental scaffolding behaviours were evaluated by behavioural observations. Data of this subset will also be included in the analysis.

Ethical considerations

This research project fulfils General Data Protection Regulation requirements for data processing, storage, and security. Ethical approval has been obtained from the Swedish Ethical

For peer review only - http://bmjopen.bmj.com/site/about/guidelines.xhtml

Review Authority (the Regional Ethical Review Board in Uppsala, reference number 2019/01170, with amendments, and reference number 2022-03146-02). Participants are informed of the study's objectives, and their data confidentiality and security are assured. Consent is obtained before each data collection, with the assurance that participants can withdraw at any time without providing a reason. Research findings will be continuously disseminated through international peer-reviewed journals, the project's website, social media channels, and national and international conferences.

Patient and public involvement

Patient and public involvement are essential to the current cohort profile. From pilot design to long-term follow-ups, a high degree of engagement from patients and the public continues to shape the development of this study. Our research team has also established open communication through a variety of platforms with the public from the beginning of the study. Study participants have been encouraged to contact the team regarding any comments and questions related to procedures. We have reviewed patients' and the public's feedback and we will continue to take this valuable information into account when examining our research approach and acquired data. The research team has close contact with several stakeholders, including clinical staff and patient organisations.

FINDINGS TO DATE

What have we learnt about peripartum depression correlates from the BASIC study?

The BASIC study aimed to identify depression and anxiety symptom patterns and their biophysiological correlates from pregnancy to 12 months postpartum (for the full publication list: https://www.basicstudie.se/publicerade-arbeten). In this cohort, we characterized five distinct trajectories of peripartum depression based on symptom onset: (a) healthy (60.6%), (b)

For peer review only

chronically depressed (14.6%), (c) depressed with early postpartum onset (10.9%), (d) depressed only in pregnancy (8.5%), and (e) depressed with late postpartum onset (5.4%) (16). Interestingly, these groups exhibited significant variations in background and psychosocial characteristics. For instance, all trajectories were associated with smoking prior to pregnancy, migraine, premenstrual mood symptoms, intimate partner violence, interpersonal trauma, negative delivery expectations, pregnancy nausea, and symphysiolysis. Early postpartum onset was linked to nulliparity, instrumental delivery, or a negative delivery experience, while early and late postpartum onset, along with chronic depression, were associated with other postpartum factors like low partner support, and bonding difficulties. Furthermore, we demonstrated that, in healthy pregnancy, most inflammatory markers increased in the postpartum period (43). Intriguingly, several markers such as monocyte chemotactic protein 4 (MCP-4) tumour necrosis factor ligand superfamily member, hepatocyte growth factor, interleukin, fibroblast growth factor 23, and C-X-C motif chemokine 1, and STAM-Binding Protein were related to different depression trajectories (44-47). Compared to healthy women, women with antenatal depression or with SSRI treatment showed lower levels of peripheral inflammatory markers (48). When examining metabolic profiles in our cohort, there were no significant depression-specific differences in untargeted gas chromatography-mass spectrometry between women with depressive symptoms and those without depressive symptoms during the antenatal phase (49). However, five metabolites (glycerol, threonine, 2-hydroxybutanoic acid, erythritol, and phenylalanine) were higher among women with postpartum depression than controls (50). Moreover, pregnancy is associated with a dysregulation of the hypothalamic-pituitary-adrenal axis and an increase in basal cortisol levels. Though the endocrine changes are prominent during pregnancy, there is no significant difference between women with depressive symptoms and those without depressive symptoms (51), the difference became significant postpartum (52). Intriguingly, among women who did

For peer review only - http://bmjopen.bmj.com/site/about/guidelines.xhtml

not have depressive symptoms during pregnancy, the levels of corticotropin-releasing hormone in the 2nd trimester were positively correlated with the levels of postpartum depressive symptoms (53). Additionally, while evidence suggests that placental glucocorticoid receptors are affected by maternal depression or anxiety (54, 55), results from one of our subsets contrast with some previous reports (56, 57). At the gene level, single nucleotide polymorphisms in the hydroxysteroid 11-beta dehydrogenase 2 gene, a key hypothalamic-pituitary-adrenal axis gene, are significantly linked to depressive symptoms in 6 weeks postpartum and the stress levels during pregnancy (58). Our data showed that women with SSRIs treatment exhibited higher levels of serotonin receptor 1A (HTR1A) and neuropeptide Y2 receptor (NPY2R) in the placenta compared to healthy women or women with untreated depression (59). Overall, the BASIC study provides evidence that can support the identification of individual risk profiles. Several psychosocial and emotional risk factors are consistent with previous literature (28). We have also gained a deeper understanding of several biological and psychological factors that are important for the clinical implications. For instance, biological factors, including corticotropin-releasing hormone (53), cortisol levels (52, 60), inflammatory/anti-inflammatory markers (43, 47, 48), stress-related genetic polymorphisms (58), and plasma metabolic profiling (50), might be related to different timing of the observed depressive symptoms which can support the diagnosis, treatment, and further pathophysiological investigation of peripartum depression. Moreover, our evidence suggests that preventive measures during delivery should be taken to avoid anemia, negative experience of delivery, and severe obstetric lacerations in order to reduce the risk for peripartum depression (61, 62). For psychological factors, our evidence suggests that when screening peripartum depression, combining evaluation of attachment and neuroticism/trait anxiety (63, 64) might facilitate early identification. Currently, several ongoing analyses focus on the integration of biopsychosocial factors using conventional statistical approaches as well as machine learning techniques. Our

For peer review only - http://bmjopen.bmj.com/site/about/guidelines.xhtml

results will provide evidence to support the development of accurate prediction as well as personalized preventive intervention and treatment.

What is the impact of peripartum depression on an infant's outcome?

The interplay between maternal well-being, peripartum exposure and childhood environment contributes to the cognitive, social and emotional development of the child (19, 20). The BASIC cohort underscored many important factors and potential underlying mechanisms behind peripartum depression. These findings have further laid the foundation to investigate the associations between peripartum depression and children's outcomes.

From the BASIC Child longitudinal subset, we observed that among mothers with lower levels of maternal postpartum depression, infants showed more gaze-following behaviour at 10 months (65). We also observed that a higher level of exposure to interpersonal traumatic events moderated the negative impact of anxiety during the 2nd trimester on an infant's attentional control from 6 to 18 months (66). In the developmental literature, gaze-following is an ability to synchronize visual attention with others (67) and this ability is linked to social learning and emotional regulation in the early years (68, 69). Attentional control in infancy has been postulated as fundamental to later learning and cognitive development (70). The negative impact of peripartum depression on infants' development was validated in our recent publication using U-BIRTH data at 18 months. We investigated the associations between different peripartum trajectories and an infant's behavioural problems at 18 months. Our results demonstrated that compared to infants of healthy mothers, infants of depressed mothers showed a higher risk of behavioural problems. Maternal antenatal and persistent depression were associated with a higher degree of behavioural problems and girls were affected to a greater degree. When we analysed the mediating effect of maternal postpartum bonding, maternal

For peer review only - http://bmjopen.bmj.com/site/about/guidelines.xhtml

persistent- and postpartum depression showed a high level of indirect effect on infants' behavioural problems, while antenatal depression had a stronger direct effect (71). We are currently investigating how maternal depression and anxiety symptoms during pregnancy are associated with infant temperament trajectories (manuscript submitted). This investigation points to a specific importance for maternal anxiety symptoms during pregnancy that are associated with infant temperament being experienced as increasingly difficult throughout the first 18 months. This is in comparison with depressive/anhedonia symptoms that are associated with infant temperament being stably medium-high in difficulty. Also, sex-specific effects were found with girls being more vulnerable towards maternal anxiety during pregnancy and boys towards maternal postpartum depression. Taken together, results from our subset (BASIC Child) and data from U-BIRTH support the hypothesis that there are negative influences of peripartum depression on an infant's outcome at the behavioural level.

At the biological level, several studies reported a significant impact of peripartum depressive symptoms on child biological and emotional outcomes. For instance, we looked at DNA methylation in the cord blood of new-borns, which is a form of pregenetic modification and most of the changes happen during embryogenesis (72). According to the foetal programming hypothesis (73), it is assumed that DNA methylation might be a possible underlying mechanism that contributes to cross-generational transmission. In our subset with 373 mother-infant dyads, we observed the differential DNA methylation in infants with mothers combined peripartum depressive symptoms and anxiety symptoms or mothers with SSRIs treatment compared to infants with healthy mothers (74). In our recent, we used DNA extracted in cord blood at birth and examined if DNA methylation differences could affect an infant's behavioural problems at 18 months while taking peripartum depression into account. However, we didn't find evidence to support the foetal programming hypothesis (75). At the physical

For peer review only - http://bmjopen.bmj.com/site/about/guidelines.xhtml

level, we investigated the relationship between prenatal depression, the mother's BMI, and infant birth weight. Large birth weight is associated with risks for several long-term adverse outcomes, such as obesity, type II diabetes, and cardiovascular disease (76). The main results of our study showed that maternal BMI during the first trimester modulates the association between antenatal depressive symptoms and birth weight. Consequently, in mothers who had a higher BMI and depressive symptoms during pregnancy, their infants were born with a greater body weight compared to infants with healthy mothers with higher BMI or depressed mothers with normal BMI (77). Previously, we also demonstrated that in non-diabetic mothers, placental gene expression in large gestational birthweight infants influences lipid metabolism, including insulin-like growth factor binding protein 1 and leptin (78). Notably, low leptin levels have been associated with postpartum depressive symptoms (79). Although the exact mechanisms linking peripartum depression, BMI, and child outcomes need further research, our findings offer valuable insights into the early identification of high-risk groups for prevention programs and facilitate a deeper understanding of the intricate relationship between depression and obesity. Furthermore, previous evidence has shown that skin-to-skin contact might help the infant regulate stress (80). However, mothers with peripartum depressive symptoms during pregnancy showed a delayed first breastfeeding session compared with mothers without depressive symptoms. Mothers who experienced depressive symptoms during pregnancy and had a delayed initiation of breastfeeding after birth continued to provide less exclusive breastfeeding at 6 weeks postpartum (81). Our results support the notion that both infants and depressed mothers might benefit from targeted breastfeeding support during the first hours after delivery.

STRENGTHS AND LIMITATIONS

For peer review only - http://bmjopen.bmj.com/site/about/guidelines.xhtml

This project has one of Scandinavian's largest longitudinal cohorts, providing comprehensive and rich data across pregnancy, infancy, childhood, and pre-teen phases. To disentangle the complex interaction between maternal well-being, peripartum exposure, and childhood environment and how they contribute to the cognitive, social and emotional development of the child, this study includes multidimensional data from biological, psychometrical, and behavioural measures. However, several limitations may limit the generalizability of the findings. Firstly, despite including a relatively large sample, the acceptance rate at the baseline of the U-BIRTH cohort from the BASIC cohort was low (ca. 50%). Secondly, from the participants' sociodemographic information, most parents enrolled hold a degree in higher education and this has been an indication of a low-risk profile (82). This might be attributed to the study being conducted in a university town, which limits the diversity of the sample. Thirdly, assessments of maternal psychometric data were primarily self-report questionnaires. This approach resulted in a lack of important objective information relating to maternal cognitive functioning and IQ, which could have an impact on, children's outcomes (83, 84). Fourthly, mothers experiencing depressive symptoms might have a higher dropout rate compared to mothers without depressive symptoms. Similarly, the cohort's representability might decrease over time due to the increased rate of missing data and decline in participation. Consequently, our data might not fully capture data on the exact severity level and the possible fluctuation of depressive symptoms over time after the peripartum period.

With regard to outcome measures, though assessments of child development included in the project are well accepted and widely applied in the field, most of them rely heavily on parent-report questionnaires. This could introduce information bias. Moreover, our study only recruited Swedish-speaking mothers. This admittedly excludes many families with an immigrant background who might have other risk factors, such as lack of social support from

For peer review only - http://bmjopen.bmj.com/site/about/guidelines.xhtml

extended family, language barriers, etc. This could also limit the generalizability of our results. Finally, our project covers a wide range of information, including socioeconomic background, living conditions, maternal health conditions, various lifestyle factors, etc. When analysing data and interpreting our results, we shall very carefully consider the potential heterogeneity present in the dataset. This entails recognizing and accounting for the diverse characteristics within our cohort, as they may influence the outcomes and conclusions drawn from our study.

Collaboration

Researchers interested in collaboration may contact the corresponding author, Alkistis Skalkidou, with their request. The U-BIRTH study steering committee considers the scientific quality of the aims and methods of incoming requests in addition to the volume of the requested data or samples. The committee prioritises study questions on peripartum depression and child development.

Authors' contributions

AS conceptualized and designed of the study. EF, TKK and MR contributed significantly to the design and execution of the project. UE and HFT contribute to the continuation of the project. HFT drafted the article, and all authors revised the draft critically for intellectual content and approved the final version.

Funding statement

This project is supported by the Uppsala Region (ALF-965886 and ALF-965905), the Marianne and Marcus Wallenberg Foundation (MMW2011.0115), the Göran Gustafsson Foundation (Nr. 1551 A-2015), and the Swedish Research Council (523-2014-07605) to AS as well as the Gillbergska Foundation to EF (year 2022).

For peer review only

Competing interest

All authors declare that they have no conflict of interest.

Acknowledgements

We are very grateful to all families who participated in this project, as well as to Carolina Wikström, Elisabeth Hansson and Amanda Olsson for administrative support.

References

1. O'Hara MW, Wisner KL. Perinatal mental illness: definition, description and aetiology. Best Practice & Research Clinical Obstetrics & Gynaecology. 2014;28(1):3-12.
2. Woody C, Ferrari A, Siskind D, Whiteford H, Harris M. A systematic review and meta-regression of the prevalence and incidence of perinatal depression. Journal of Affective Disorders. 2017;219:86-92.
3. Underwood L, Waldie K, D'Souza S, Peterson ER, Morton S. A review of longitudinal studies on antenatal and postnatal depression. Archives of Women's Mental Health. 2016;19(5):711-20.
4. Gavin NI, Gaynes BN, Lohr KN, Meltzer-Brody S, Gartlehner G, Swinson T. Perinatal depression: a systematic review of prevalence and incidence. Obstetrics & Gynecology. 2005;106(5 Part 1):1071-83.
5. Mitchell AR, Gordon H, Lindquist A, Walker SP, Homer CS, Middleton A, et al. Prevalence of Perinatal Depression in Low-and Middle-Income Countries: A Systematic Review and Meta-analysis. JAMA Psychiatry. 2023.
6. Healey C, Morriss R, Henshaw C, Wadoo O, Sajjad A, Scholefield H, et al. Self-harm in postpartum depression and referrals to a perinatal mental health team: an audit study. Archives of Women's Mental Health. 2013;16(3):237-45.
7. Lindahl V, Pearson JL, Colpe L. Prevalence of suicidality during pregnancy and the postpartum. Archives of Women's Mental Health. 2005;8(2):77-87.
8. Cusick S, Georgieff MK. The first 1,000 days of life: The brain's window of opportunity. New York, NY: UNICEF. 2016.
9. Mwaniki MK, Atieno M, Lawn JE, Newton CR. Long-term neurodevelopmental outcomes after intrauterine and neonatal insults: a systematic review. The Lancet. 2012;379(9814):445-52.
10. Huot R, Brennan P, Stowe Z, Plotsky P, Walker E. Negative affect in offspring of depressed mothers is predicted by infant cortisol levels at 6 months and maternal depression during pregnancy, but not postpartum. Annals of the New York Academy of Sciences. 2004;1032(1):234-6.
11. Power J, van IJzendoorn M, Lewis AJ, Chen W, Galbally M. Maternal perinatal depression and child executive function: A systematic review and meta-analysis. Journal of Affective Disorders. 2021;291:218-34.

For peer review only - http://bmjopen.bmj.com/site/about/guidelines.xhtml

12. Nidey NL, Momany AM, Strathearn L, Carter KD, Wehby GL, Bao W, et al. Association between perinatal depression and risk of attention deficit hyperactivity disorder among children: A retrospective cohort study. Annals of Epidemiology. 2021;63:1-6.
13. Chen L-C, Chen M-H, Hsu J-W, Huang K-L, Bai Y-M, Chen T-J, et al. Association of parental depression with offspring attention deficit hyperactivity disorder and autism spectrum disorder: A nationwide birth cohort study. Journal of Affective Disorders. 2020;277:109-14.
14. Tirumalaraju V, Suchting R, Evans J, Goetzl L, Refuerzo J, Neumann A, et al. Risk of depression in the adolescent and adult offspring of mothers with perinatal depression: a systematic review and meta-analysis. JAMA Network Open. 2020;3(6):e208783-e.
15. Rogers A, Obst S, Teague SJ, Rossen L, Spry EA, Macdonald JA, et al. Association between maternal perinatal depression and anxiety and child and adolescent development: a meta-analysis. JAMA Pediatrics. 2020;174(11):1082-92.
16. Wikman A, Axfors C, Iliadis SI, Cox J, Fransson E, Skalkidou A. Characteristics of women with different perinatal depression trajectories. Journal of Neuroscience Research. 2020;98(7):1268-82.
17. Keim SA, Daniels JL, Dole N, Herring AH, Siega-Riz AM, Scheidt PC. A prospective study of maternal anxiety, perceived stress, and depressive symptoms in relation to infant cognitive development. Early Human Development. 2011;87(5):373-80.
18. Axfors C, Bränn E, Henriksson HE, Hellgren C, Kallak TK, Fransson E, et al. Cohort profile: the Biology, Affect, Stress, Imaging and Cognition (BASIC) study on perinatal depression in a population-based Swedish cohort. BMJ Open. 2019;9(10):e031514.
19. Kingston D, Tough S, Whitfield H. Prenatal and postpartum maternal psychological distress and infant development: a systematic review. Child Psychiatry & Human Development. 2012;43(5):683-714.
20. Stein A, Pearson RM, Goodman SH, Rapa E, Rahman A, McCallum M, et al. Effects of perinatal mental disorders on the fetus and child. The Lancet. 2014;384(9956):1800-19.
21. Van Steijn DJ, Oerlemans AM, Van Aken MA, Buitelaar JK, Rommelse NN. The reciprocal relationship of ASD, ADHD, depressive symptoms and stress in parents of children with ASD and/or ADHD. Journal of Autism and Developmental Disorders. 2014;44(5):1064-76.
22. Silva-Fernandes A, Conde A, Marques M, Caparros-Gonzalez RA, Fransson E, Mesquita AR, et al. Inflammatory biomarkers and perinatal depression: a systematic review. medRxiv. 2023:2023.01. 11.23284231.
23. Luo F, Zhu Z, Du Y, Chen L, Cheng Y. Risk Factors for Postpartum Depression Based on Genetic and Epigenetic Interactions. Molecular Neurobiology. 2023;60(7):3979-4003.
24. Serati M, Redaelli M, Buoli M, Altamura A. Perinatal major depression biomarkers: a systematic review. Journal of Affective Disorders. 2016;193:391-404.
25. Wang L, Zhang M, Zhu H, Sun L, Yu B, Cui X. Combined identification of lncRNA NONHSAG004550 and NONHSAT125420 as a potential diagnostic biomarker of perinatal depression. Journal of Clinical Laboratory Analysis. 2021;35(8):e23890.
26. Guintivano J, Arad M, Gould TD, Payne JL, Kaminsky ZA. Antenatal prediction of postpartum depression with blood DNA methylation biomarkers. Molecular Psychiatry. 2014;19(5):560-7.
27. Li S, Yang Z, Yao M, Shen Y, Zhu H, Jiang Y, et al. Exploration for biomarkers of postpartum depression based on metabolomics: A systematic review. Journal of Affective Disorders. 2022.
28. Hutchens BF, Kearney J. Risk factors for postpartum depression: an umbrella review. Journal of Midwifery & Women's Health. 2020;65(1):96-108.

For peer review only - http://bmjopen.bmj.com/site/about/guidelines.xhtml

29. Lawlor DA, Leary S, Smith GD. Theoretical underpinning for the use of intergenerational studies in life course epidemiology. Family Matters: Designing, Analysing and Understanding Family Based Studies in Life Course Epidemiology. 2009:13-38.

30. APA. DSM-5. American Psychiatric Association. 2013.

31. Maruish ME. User's manual for the SF-36v2 Health Survey: Quality Metric Incorporated; 2011.

32. Group TE. EuroQol-a new facility for the measurement of health-related quality of life. Health Policy. 1990;16(3):199-208.

33. Dumas JE, Nissley J, Nordstrom A, Smith EP, Prinz RJ, Levine DW. Home chaos: Sociodemographic, parenting, interactional, and child correlates. Journal of Clinical Child and Adolescent Psychology. 2005;34(1):93-104.

34. Haskett ME, Ahern LS, Ward CS, Allaire JC. Factor structure and validity of the parenting stress index-short form. Journal of Clinical Child & Adolescent Psychology. 2006;35(2):302-12.

35. Östberg M, Hagekull B. Frågeformulär angående föräldrapåfrestning (The Swedish Parenthood Stress Questionnaire-SPSQ) Beskrivning av instrumentets konstruktion, normdata, reliabilitet och validitet. Opublicerat manuskript, Uppsala Universitet, Sverige; 2012.

36. Putnam SP, Gartstein MA, Rothbart MK. Measurement of fine-grained aspects of toddler temperament: The Early Childhood Behavior Questionnaire. Infant Behavior and Development. 2006;29(3):386-401.

37. Achenbach TM, Rescorla LA. Manual for the ASEBA preschool forms and profiles: Burlington, VT: University of Vermont, Research center for children, youth …; 2000.

38. Achenbach TM. Manual for ASEBA school-age forms & profiles. University of Vermont, Research Center for Children, Youth & Families. 2001.

39. Rescorla L, Alley A. Validation of the language development survey (LDS). 2001.

40. Kadesjö B, Janols L-O, Korkman M, Mickelsson K, Strand G, Trillingsgaard A, et al. The FTF (Five to Fifteen): the development of a parent questionnaire for the assessment of ADHD and comorbid conditions. European Child & Adolescent Psychiatry. 2004;13(3):iii3-iii13.

41. Korkman M, Kadesjö B, Trillingsgaard A, Mickelsson K, Janols L-O, Strand G, et al. Frågeformulär för utredning av utveckling och beteende hos 5 till 15 år gamla barn. 2005.

42. Gredebäck G, Forssman L, Lindskog M, Kenward B. The BasicChild Project. 2019.

43. Bränn E, Edvinsson Å, Rostedt Punga A, Sundström-Poromaa I, Skalkidou A. Inflammatory and anti-inflammatory markers in plasma: from late pregnancy to early postpartum. Scientific Reports. 2019;9(1):1-10.

44. Osimo EF, Pillinger T, Rodriguez IM, Khandaker GM, Pariante CM, Howes OD. Inflammatory markers in depression: a meta-analysis of mean differences and variability in 5,166 patients and 5,083 controls. Brain, Behavior, and Immunity. 2020;87:901-9.

45. Henriksson HE, White RA, Iliadis SI, Fransson E, Papadopoulos FC, Sundström-Poromaa I, et al. Spring peaks and autumn troughs identified in peripheral inflammatory markers during the peripartum period. Scientific Reports. 2019;9(1):1-13.

46. Bränn E, Fransson E, White RA, Papadopoulos FC, Edvinsson Å, Kamali-Moghaddam M, et al. Inflammatory markers in women with postpartum depressive symptoms. Journal of Neuroscience Research. 2020;98(7):1309-21.

47. Bränn E, Papadopoulos F, Fransson E, White R, Edvinsson Å, Hellgren C, et al. Inflammatory markers in late pregnancy in association with postpartum depression—a nested case-control study. Psychoneuroendocrinology. 2017;79:146-59.

For peer review only - http://bmjopen.bmj.com/site/about/guidelines.xhtml

48. Edvinsson Å, Bränn E, Hellgren C, Freyhult E, White R, Kamali-Moghaddam M, et al. Lower inflammatory markers in women with antenatal depression brings the M1/M2 balance into focus from a new direction. Psychoneuroendocrinology. 2017;80:15-25.
49. Henriksson HE, Malavaki C, Bränn E, Drainas V, Lager S, Iliadis SI, et al. Blood plasma metabolic profiling of pregnant women with antenatal depressive symptoms. Translational Psychiatry. 2019;9(1):1-9.
50. Bränn E, Malavaki C, Fransson E, Ioannidi M-K, Henriksson HE, Papadopoulos FC, et al. Metabolic Profiling Indicates Diversity in the Metabolic Physiologies Associated With Maternal Postpartum Depressive Symptoms. Frontiers in Psychiatry. 2021;12.
51. Hellgren C, Åkerud H, Skalkidou A, Sundström-Poromaa I. Cortisol awakening response in late pregnancy in women with previous or ongoing depression. Psychoneuroendocrinology. 2013;38(12):3150-4.
52. Iliadis SI, Comasco E, Sylvén S, Hellgren C, Sundström Poromaa I, Skalkidou A. Prenatal and postpartum evening salivary cortisol levels in association with peripartum depressive symptoms. PloS One. 2015;10(8):e0135471.
53. Iliadis SI, Sylvén S, Hellgren C, Olivier JD, Schijven D, Comasco E, et al. Mid-pregnancy corticotropin-releasing hormone levels in association with postpartum depressive symptoms. Depression and Anxiety. 2016;33(11):1023-30.
54. Togher KL, Treacy E, O'Keeffe GW, Kenny LC. Maternal distress in late pregnancy alters obstetric outcomes and the expression of genes important for placental glucocorticoid signalling. Psychiatry Research. 2017;255:17-26.
55. Reynolds R, Pesonen A-K, O'reilly J, Tuovinen S, Lahti M, Kajantie E, et al. Maternal depressive symptoms throughout pregnancy are associated with increased placental glucocorticoid sensitivity. Psychological Medicine. 2015;45(10):2023-30.
56. Edvinsson Å, Hoyer A, Hansson M, Kallak TK, Sundström-Poromaa I, Skalkidou A, et al. Placental glucocorticoid receptors are not affected by maternal depression or SSRI treatment. Upsala Journal of Medical Sciences. 2020;125(1):30-6.
57. Hellgren C, Comasco E, Skalkidou A, Sundström-Poromaa I. Allopregnanolone levels and depressive symptoms during pregnancy in relation to single nucleotide polymorphisms in the allopregnanolone synthesis pathway. Hormones and Behavior. 2017;94:106-13.
58. Skalkidou A, Poromaa IS, Iliadis SI, Huizink AC, Hellgren C, Freyhult E, et al. Stress-related genetic polymorphisms in association with peripartum depression symptoms and stress hormones: a longitudinal population-based study. Psychoneuroendocrinology. 2019;103:296-305.
59. Edvinsson Å, Hellgren C, Kunovac Kallak T, Åkerud H, Skalkidou A, Stener-Victorin E, et al. The effect of antenatal depression and antidepressant treatment on placental tissue: a protein-validated gene expression study. BMC Pregnancy and Childbirth. 2019;19(1):1-13.
60. Hellgren C, Edvinsson Å, Olivier JD, Fornes R, Stener-Victorin E, Ubhayasekera SKA, et al. Tandem mass spectrometry determined maternal cortisone to cortisol ratio and psychiatric morbidity during pregnancy− interaction with birth weight. Psychoneuroendocrinology. 2016;69:142-9.
61. Eckerdal P, Kollia N, Löfblad J, Hellgren C, Karlsson L, Högberg U, et al. Delineating the association between heavy postpartum haemorrhage and postpartum depression. PloS One. 2016;11(1):e0144274.
62. Asif S, Mulic-Lutvica A, Axfors C, Eckerdal P, Iliadis SI, Fransson E, et al. Severe obstetric lacerations associated with postpartum depression among women with low resilience–a Swedish birth cohort study. BJOG: An International Journal of Obstetrics & Gynaecology. 2020;127(11):1382-90.

For peer review only - http://bmjopen.bmj.com/site/about/guidelines.xhtml

63. Axfors C, Sylvén S, Ramklint M, Skalkidou A. Adult attachment's unique contribution in the prediction of postpartum depressive symptoms, beyond personality traits. Journal of Affective Disorders. 2017;222:177-84.
64. Iliadis SI, Comasco E, Hellgren C, Kollia N, Poromaa IS, Skalkidou A. Associations between a polymorphism in the hydroxysteroid (11-beta) dehydrogenase 1 gene, neuroticism and postpartum depression. Journal of Affective Disorders. 2017;207:141-7.
65. Astor K, Lindskog M, Forssman L, Kenward B, Fransson M, Skalkidou A, et al. Social and emotional contexts predict the development of gaze following in early infancy. Royal Society Open Science. 2020;7(9):201178.
66. Tu H-F, Skalkidou A, Lindskog M, Gredebäck G. Maternal childhood trauma and perinatal distress are related to infants' focused attention from 6 to 18 months. Scientific Reports. 2021;11(1):24190.
67. Driver IV J, Davis G, Ricciardelli P, Kidd P, Maxwell E, Baron-Cohen S. Gaze perception triggers reflexive visuospatial orienting. Visual Cognition. 1999;6(5):509-40.
68. Brooks R, Meltzoff AN. Connecting the dots from infancy to childhood: A longitudinal study connecting gaze following, language, and explicit theory of mind. Journal of Experimental Child Psychology. 2015;130:67-78.
69. Mundy P. Annotation: The neural basis of social impairments in autism: The role of the dorsal medial-frontal cortex and anterior cingulate system. Journal of Child Psychology and Psychiatry. 2003;44(6):793-809.
70. Posner MI, Rothbart MK, Sheese BE, Voelker P. Developing attention: behavioral and brain mechanisms. Advances in Neuroscience. 2014;2014.
71. Fransson E, Sörensen F, Kallak TK, Ramklint M, Eckerdal P, Heimgärtner M, et al. Maternal perinatal depressive symptoms trajectories and impact on toddler behavior–the importance of symptom duration and maternal bonding. Journal of Affective Disorders. 2020;273:542-51.
72. Reik W. Stability and flexibility of epigenetic gene regulation in mammalian development. Nature. 2007;447(7143):425-32.
73. Barker DJP. The developmental origins of adult disease. Journal of the American College of Nutrition. 2004;23(sup6):588S-95S.
74. Kallak TK, Bränn E, Fransson E, Johansson Å, Lager S, Comasco E, et al. DNA methylation in cord blood in association with prenatal depressive symptoms. Clinical Epigenetics. 2021;13(1):1-14.
75. Kallak TK, Fransson E, Bränn E, Berglund H, Lager S, Comasco E, et al. Maternal prenatal depressive symptoms and toddler behavior: an umbilical cord blood epigenome-wide association study. Translational Psychiatry. 2022;12(1):1-11.
76. Scifres CM. Short-and long-term outcomes associated with large for gestational age birth weight. Obstetrics and Gynecology Clinics. 2021;48(2):325-37.
77. Petursdottir Maack H, Skalkidou A, Sjöholm A, Eurenius-Orre K, Mulic-Lutvica A, Wikström A-K, et al. Maternal body mass index moderates antenatal depression effects on infant birthweight. Scientific Reports. 2019;9(1):1-7.
78. Ahlsson F, Åkerud H, Schijven D, Olivier J, Sundström-Poromaa I. Gene expression in placentas from nondiabetic women giving birth to large for gestational age infants. Reproductive Sciences. 2015;22(10):1281-8.
79. Skalkidou A, Sylvén SM, Papadopoulos FC, Olovsson M, Larsson A, Sundström-Poromaa I. Risk of postpartum depression in association with serum leptin and interleukin-6 levels at delivery: A nested case–control study within the UPPSAT cohort. Psychoneuroendocrinology. 2009;34(9):1329-37.

For peer review only - http://bmjopen.bmj.com/site/about/guidelines.xhtml

80. Ionio C, Ciuffo G, Landoni M. Parent–infant skin-to-skin contact and stress regulation: A systematic review of the literature. International Journal of Environmental Research and Public Health. 2021;18(9):4695.
81. Cato K, Sylvén SM, Georgakis MK, Kollia N, Rubertsson C, Skalkidou A. Antenatal depressive symptoms and early initiation of breastfeeding in association with exclusive breastfeeding six weeks postpartum: a longitudinal population-based study. BMC Pregnancy and Childbirth. 2019;19(1):1-11.
82. Lancaster CA, Gold KJ, Flynn HA, Yoo H, Marcus SM, Davis MM. Risk factors for depressive symptoms during pregnancy: a systematic review. American Journal of Obstetrics and Gynecology. 2010;202(1):5-14.
83. Lean RE, Paul RA, Smyser CD, Rogers CE. Maternal intelligence quotient (IQ) predicts IQ and language in very preterm children at age 5 years. Journal of Child Psychology and Psychiatry. 2018;59(2):150-9.
84. Ye A, Yan S, Huang K, Mao L, Ge X, Weng T, et al. Maternal intelligence quotient and motor development in early childhood: The mediating role of mother's education. Journal of Paediatrics and Child Health. 2019;55(1):87-94.

For peer review only

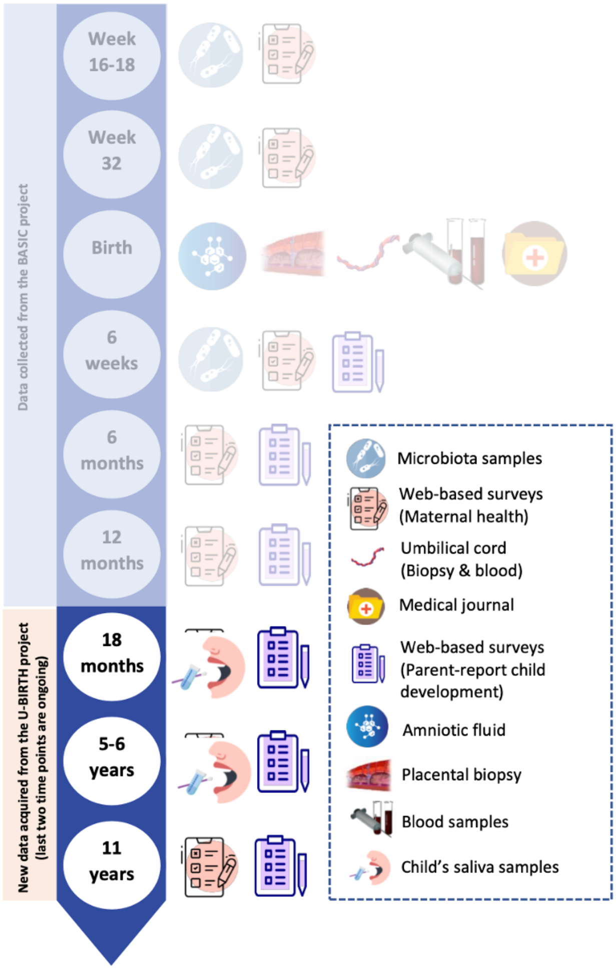

Figure 1. U-BIRTH study timeline

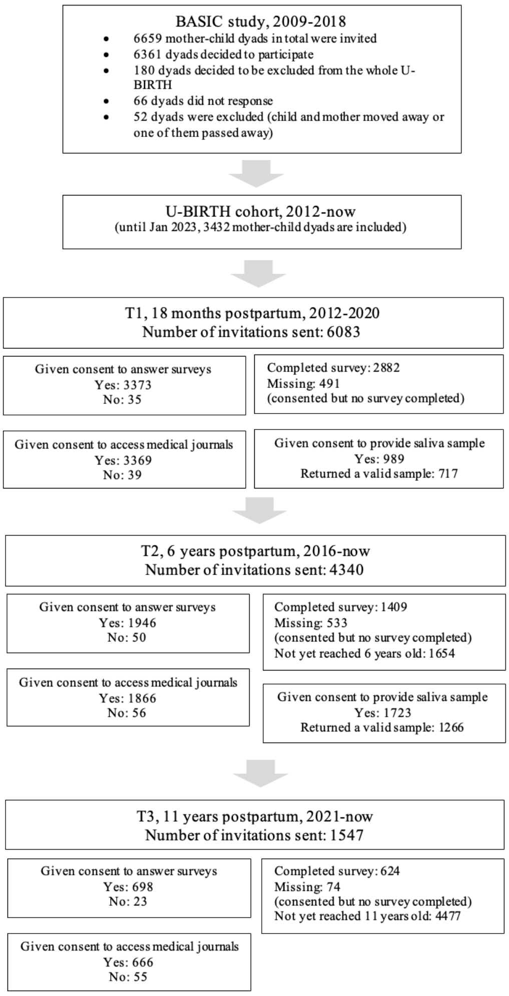

Figure 2. Flowchart of the U-BIRTH study participants

For peer review only

Supplementary Table 1. Content of maternal surveys included in the BASIC and the U-BIRTH studies

**Domain of measure**	**Gestational week 16–18**	**Gestational week 32**	**Child-birth**	**Postpartum 6 weeks**	**Postpartum 6 months**	**Postpartum 12 months**	**Postpartum 18 months**	**Postpartum 6 years**	**Postpartum 11 years**
	**Data collected from the BASIC project (all completed)**						**Data collected from the U-BIRTH project (Data from 18 months are completed; data collection for 6 and 11 years is ongoing)**		
Depressive symptoms	EPDS<br>DSRS<br>History of depression	EPDS	EPDS	EPDS<br>DSRS (2011–)	EPDS<br>DSRS (2011–)	Single item "how are you"?	DSRS	PHQ-9	PHQ-9<br>DSM-5 Self-Rated Level 1 CCSM
Partner's depressive symptoms				Single item "partner's mental health"					
Anxiety symptoms	BAI (2013–)<br>EPDS†<br>STAI-S (2011–2012)	BAI (2013–)<br>EPDS†<br>STAI-S(2011–2012)<br>STAI-T (2011–2012)		BAI (2013–)<br>EPDS†<br>STAI-S (2011–2012)	BAI (2013–)<br>EPDS†<br>STAI-S (2011–2012)<br>STAI-T (2011–2012)				
Trauma and stressful events	Intimate partner violence*			SLE<br>SQ-PTSD (–2011)	SLE<br>SQ-PTSD (2011–)	LITE (2013–)	SLE<br>SPSQ	New SLE	New SLE
Obstetric and gynecological information	Planning of pregnancy<br>Nausea/vomiting<br>Premenstrual symptoms	Pregnancy complications<br>Fear of delivery<br>Delivery expectation		Delivery information<br>Delivery experience					
Medical information	Depression history*<br>Prior contact with psychiatry*<br>Somatic health conditions*<br>Prior and current medication*	Current medication*<br>RLS			Current medication*	Current medication* (2013–)	Current medication*<br>Medical journal	Current medication*<br>Medical journal	Current medication*<br>Medical journal
Lifestyle	BMI before pregnancy*<br>Alcohol*<br>Smoking*<br>Tobacco*	BMI<br>Diet*<br>Alcohol*<br>Smoking*<br>Tobacco*		BMI<br>Smoking*<br>Alcohol*<br>Tobacco*	Smoking*<br>Alcohol*<br>Tobacco*		Smoking*<br>Alcohol*<br>Tobacco*	Smoking*<br>Alcohol*<br>Tobacco*	Smoking*<br>Alcohol*<br>Tobacco*

For peer review only

Supplementary Table 1. (Continued)

Domain of measure	Gestational week 16–18	Gestational week 32	Child-birth	Postpartum 6 weeks	Postpartum 6 months	Postpartum 12 months	Postpartum 18 months	Postpartum 6 years	Postpartum 11 years
Sleep	Sleeping habits before pregnancy*<br>Hours of sleep during pregnancy*<br>Sleep quality (2018–)*	Sleeping problems*<br>Hours of sleep during pregnancy*<br>Sleep quality (2018–)*		Sleep quality*	Sleep quality*	Hours of sleep*	Sleep quality*	Sleep quality*	Sleep quality*
Personality and resilience measures		ASQ<br>SSP (–2011)<br>SOC-29 (2013–)<br>RS-14 (2013–)			PBQ	VPSQ (2013–)			SOC
Others	Sociodemographic information: age, , country of birth, education, employment*	Sociodemographic information: employment*		Breastfeeding*<br>General questions*<br>Life right now*<br>Partner support*<br>Social support from others*	Breastfeeding*<br>General questions*<br>Life right now*Marital status* Partner support*	Breastfeeding*<br>General questions*<br>Life right now (2013–)*	CHAOS<br>Chores and activity*<br>Life right now*<br>PSS<br>SF-36<br>Second parent's status*<br>Social life*	Another pregnancy*<br>CHAOS<br>Chores and activities*Life right now*<br>Maternal leave*<br>Partner's status*<br>Previous breastfeeding*<br>PSI<br>SF-36<br>Social life*<br>Sociodemographic information*	CHAOS<br>EQ-5<br>PSQ<br>Sociodemographic information*

ASQ: Attachment Style Questionnaire; BAI: Beck Anxiety Inventory; CCSM: Cross-Cutting Symptom measure, DSM-5; CHAOS: Confusion, Hubbub and Order Scale ; DSRS: Depression Self-Rating Scale; DSM-5: the Diagnostic and Statistical Manual of Mental Disorders, the 5th Edition; EPDS: Edinburgh Postnatal Depression Scale; EQ-5D: Health-Related Quality of Life Questionnaire; LITE: Lifetime Incidence of Traumatic Events; PBQ: Postpartum Bonding Questionnaire; PHQ9: Patient Health Questionnaire; PSI: Parenting Stress Index; PSQ: Parenting Style Questionnaire; PSS: Perceived Stress Scale; RLS: Restless legs questionnaire; RS-14: Resilience Scale; SF-36: The 36-Item Short Form Health Survey; SLE: Stressful Life Events Scale; SOC-29: Sense of Coherence; SPSQ: Swedish Parenthood Stress Questionnaire; SQ-PTSD: Screen Questionnaire-Post-Traumatic Stress Disorder; SSP: Swedish Universities Scales of Personality; STAI-S: State-Trait Anxiety Inventory-State Scale; STAI-T: State-Trait Anxiety Inventory-Trait Scale; VPSQ: Vulnerable Personality Style Questionnaire. Period of usage in the BASIC is stated in parentheses if other than 2009–2017. *Questions designed by the research team. †The Edinburgh Postnatal Depression subscales for detecting anxiety.

For peer review only
